# Neural Circuit Mapping and Neurotherapy-Based Strategies

**DOI:** 10.1007/s10571-025-01595-5

**Published:** 2025-07-26

**Authors:** Hany E. Marei

**Affiliations:** Department of Cytology and Histology, Faculty of Veterinary Medicine, Mansoura University, Mansoura, 35116 Egypt

**Keywords:** Neural circuit mapping, Neurotherapy, Brain connectivity, Neuroplasticity, Neuromodulation, Neural regeneration, Therapeutic interventions

## Abstract

Recent developments in neural circuit mapping and neurotherapy are changing our understanding of the dynamic network structure of the brain and offering new treatment options. In many neurological and psychiatric diseases, targeted control of specific brain circuits has proven to be a successful strategy to reduce cognitive, behavioral, and motor abnormalities. Sophisticated retrograde tracing techniques, transcranial magnetic stimulation (TMS), chemogenetics, optogenetics, and other technologies have greatly improved our ability to outline, observe, and control neuronal circuits with remarkable accuracy. These sophisticated techniques have revealed crucial information on neuroplasticity, circuit remodeling following injury, and the therapeutic potential of neuromodulatory interventions. Disorders include depression, anxiety, stroke, and neurodegenerative diseases are treated using techniques such as optogenetic stimulation, chemogenetic activation, and non-invasive brain stimulation to restore circuit function. Emerging multifunctional probes like Tetracysteine Display of Optogenetic Elements (Tetro-DOpE) provide real-time monitoring and modification of neuronal populations, improving circuit-level interventions’ precision. At the same time, especially following severe brain injury and neurodegeneration, stem cell treatments combined with neurogenesis-promoting strategies show great promise in increasing circuit repair and functional recovery. The development of drug delivery methods like tailored nanoparticle systems and multifunctional probes is helping to improve the accuracy and safety of treatments by reducing off-target effects. These developments taken together draw attention to a notable shift toward precision neuromedicine. These techniques are meant to offer more efficient, focused, and specialized treatments for various neurological and psychiatric diseases by combining sophisticated circuit mapping with tailored therapeutic interventions.

## Introduction

Creating tailored neurotherapeutic strategies and clarifying the concepts of brain function depend on understanding the anatomical and functional structure of neural networks. The complex interaction of specialized neural circuits underlies cognitive functions, memory consolidation, and motor coordination; disturbances in these networks are linked to the pathophysiology of several neurological and neuropsychiatric diseases including depression, AD, epilepsy, and schizophrenia.

Recent developments in neural circuit mapping technologies—such as optogenetics, chemogenetics, viral tracing, and advanced retrograde and anterograde labeling techniques—have allowed unmatched accuracy in delineating the connectivity and functional relevance of neural networks (Deisseroth 2011; Luo et al. 2018). While chemogenetics uses engineered receptors triggered by synthetic drugs to exactly modify neural activity, optogenetics allows exact temporal control of genetically targeted neurons via light-sensitive ion channels (Roth 2016). Viral tracing techniques such as monosynaptic rabies virus systems and adeno-associated virus (AAV)-based tracers also offer exact mapping of circuit inputs and outputs at single-synapse accuracy.

Its fundamental role in spatial cognition and memory as well as its link to neuropsychiatric diseases including AD and schizophrenia has drawn great attention to the retrosplenial cortex (RSC) (Maguire 2001; Vann et al. 2009). Recent studies have found distinct RSC circuits projecting to the anterodorsal thalamus (AD) and the secondary motor cortex (M2), showing intricate input and output connection patterns. While AD-projecting neurons are mostly affected by the medial septum and anterior cingulate cortex, advanced viral tracing revealed that M2-projecting RSC neurons mostly get inputs from the dorsal subiculum, anterior dorsal thalamus, lateral dorsal thalamus, lateral posterior thalamus, and somatosensory cortex. While the suppression of AD-projecting neurons mostly impairs spatial memory, functional probing with optogenetic silencing showed that the inhibition of M2-projecting RSC neurons inhibits object-location memory and action planning (Mao et al. 2017). These results highlight the semi-autonomous operation of parallel RSC pathways and offer understanding of circuit-specific vulnerabilities that could be therapeutically targeted in cognitive diseases.

**Fig. 1:**
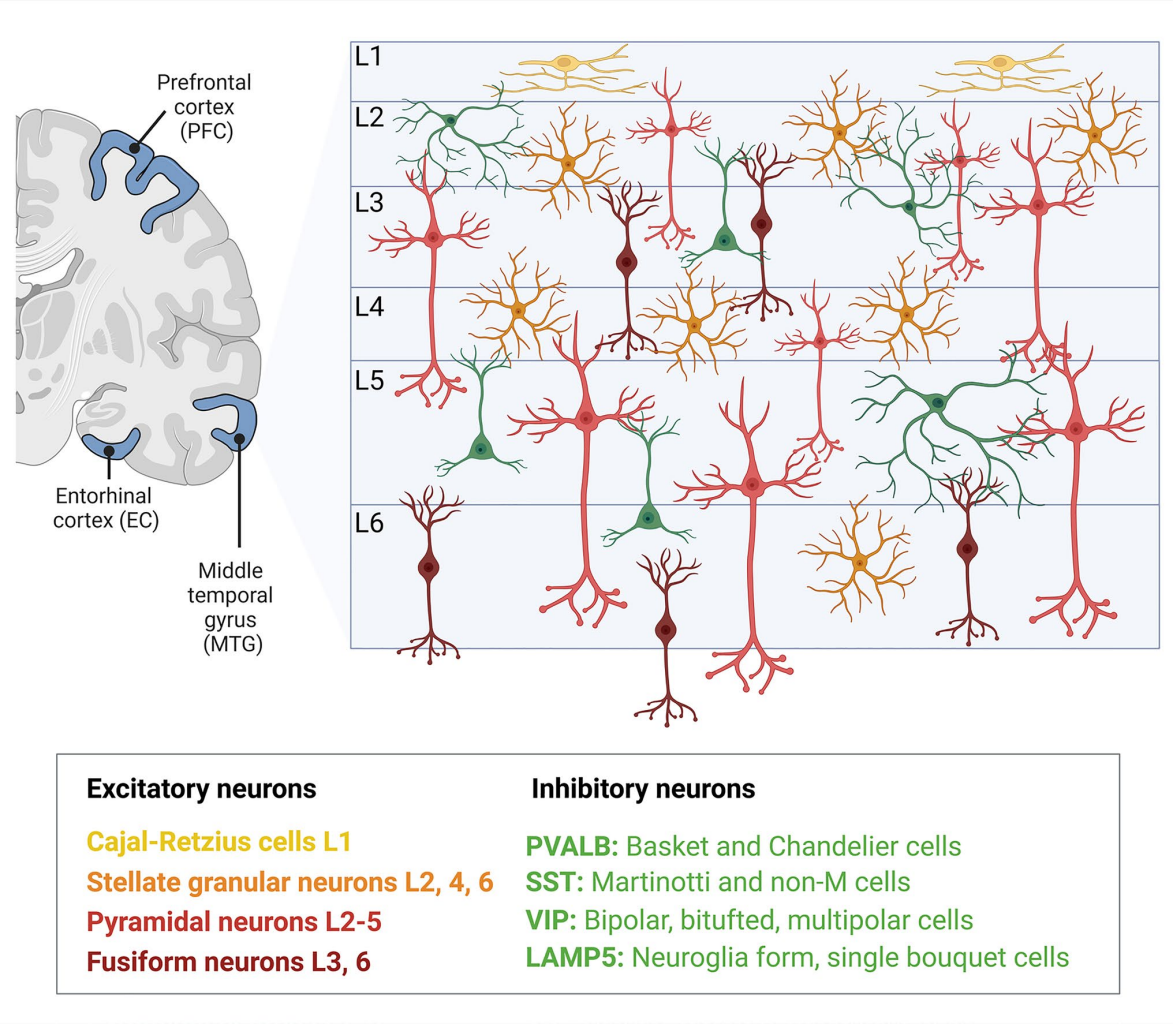
Human cortical layers’ excitatory and inhibitory neuron distribution Layers of the human cortex: excitatory and inhibitory neuron distribution. The six cortical layers (L1–L6) in the middle temporal gyrus (MTG), entorhinal cortex (EC), and prefrontal cortex (PFC) are shown in the diagram together with excitatory and inhibitory neurons distribution. Among excitatory neurons are cajal-retains cells (L1), stellate granular neurons (L2, 4, and 6), pyramidal neurons (L2, 5), and fusiform neurons (L3 and 6). The several subtypes of inhibitory neurons are PvalB (which comprises basket and chandelier cells), SST (which includes Martinotti and non-M cells), VIP (which comprises bipolar, tufted, and multipolar cells), and LAMP5 (which includes neuroglia form and single bouquet cells)

The complex dynamics of information processing in the brain are regulated by excitatory and inhibitory neurons. While guaranteeing balance to prevent network hyperexcitability, excitatory neurons like pyramidal neurons, stellate cells, Cajal-Retzius cells, and fusiform neurons serve as the main signal transmission facilitators (Treble et al. 2016). Excitatory transmission, timing, and synchronization of cortical activity are modulated by inhibitory interneurons such as parvalbumin (Pvalb)-positive basket and chandelier cells, somatostatin (SST)-positive Martinotti cells, vasoactive intestinal peptide (VIP)-positive bipolar cells, and LAMP5-expressing neurogliaform cells (Fig. 1) (Kepecs and Fishell 2014; Tremblay et al. 2016). Conditions like epilepsy, schizophrenia, and autism spectrum disorders are notably related to the disruption of inhibitory circuits (Huang and Paul 2019; Sohal and Rubenstein 2019).

Mapping of hippocampus circuits required for memory processing and spatial navigation has revealed basic ideas of network organization. Excitatory neurons in the dorsal hippocampus CA1 area got balanced input from the subiculum complex and the entorhinal cortex (EC), showing varied connectivity along the proximal–distal axis, according to monosynaptic rabies virus tracing (Sun et al. 2014). Significantly, CA3 inputs to proximal CA1 were demonstrated to be relatively stronger than those to distal regions. Furthermore, different input gradients from the subiculum and presubiculum affect the connectivity model by means of subiculum inputs increasing and presubiculum inputs decreasing along the proximal–distal CA1 axis (Sun et al. 2014). Functional studies show that these particular input streams are necessary for encoding spatial memory and contextual information, which are impaired in AD, epilepsy, and related disorders.

Emphasizing their relevance for understanding and treating neuropsychiatric and neurological disorders, this review article combines recent neurotherapeutic technology and brain circuit mapping developments. We explore how targeted neuromodulatory treatments—such as optogenetic stimulation, chemogenetic modulation, and advanced viral tracking—can examine and therapeutically alter certain neuronal networks. A thorough knowledge of cortical and hippocampal network structures will enable precision medicine approaches that tailor treatments for circuit-specific problems, hence transforming the management of cognitive and behavioral disorders.

## Neural Circuits and Function

### Viral Tracing, Optogenetics, and Chemogenetics Applied for Brain Circuit Mapping

The inclusion of nanostructured photonic probes into neuroscience marks a revolutionary shift in the analysis and therapeutic modification of brain circuitry. Taha et al. (2024) showed that the combination of nanotechnology and photonics enables precise control and high-fidelity observation of brain activity at both cellular and subcellular levels. Using tailored optogenetic stimulation and ultra-high-resolution recordings, these sophisticated nanostructured sensors can change and track brain circuitry in real time. Remarkable spatial (~ 100 nm) and temporal (~ ms) accuracy produced by the unique optical and material properties of nanomaterials surpasses that of traditional electrodes and fiber-based systems (Pisanello et al. 2017; Taha et al. 2024). These possibilities include customized stimulation for epilepsy and psychiatric disorders and establish new ones for fundamental neuroscience, as well as the development of minimally invasive neurotherapeutic techniques. Nanostructured photonic probes’ versatility allows integration with brain-machine interfaces, real-time neurochemical monitoring, and optogenetic control (Canales et al. 2018; Taha et al. 2024).

Notwithstanding their significant technical benefits, Taha et al. (2024) emphasize the need to study the relationship between nanophotonic devices and changing brain networks. Understanding brain activity during developmental stages is important to avoid maladaptive restructuring and to use therapeutic plasticity. Dooley and van der Heijden (2024) suggest a “developmental systems neuroscience” model in which circuit formation, maturation, and reconfiguration studies reveal vital intervention moments. They argue that brain networks are necessary for establishing functional architecture and go through transient activity states that sometimes appear ‘abnormal’ compared to the adult brain. Poor navigation of these transitional phases is linked to neurodevelopmental diseases, including autism spectrum disorder and schizophrenia (Stiles and Jernigan 2010; Dooley and van der Heijden 2024). Researchers can more precisely find therapy windows and create age-appropriate therapies by defining developmental circuit dynamics.

Apart from developmental elements, the functional integration of brain subcircuits supporting essential physiological activities has to be defined. Oliveira et al. (2024) investigate this theory by thorough anatomical and functional mapping of the postinspiratory complex (PiCo), a brainstem nucleus vital for controlling respiration and autonomic responses. Using viral tracing techniques—specifically adeno-associated virus (AAV) vectors and monosynaptic rabies virus, they showed that PiCo integrates several afferent inputs from nuclei, including the intermediate reticular nucleus (IRt), nucleus of the solitary tract (NTS), and A1/C1 catecholaminergic neurons. PiCo also innervates vital respiratory centers, such as the contralateral PiCo and the pre-Bötzinger complex (preBötC), hence emphasizing its key role in the generation of respiratory rhythm and autonomic integration (Anderson et al. 2016; Oliveira et al. 2024).

The growing understanding of circuit integration goes beyond somatic motor and autonomic systems. Using a spectrum of retrograde tracers—e.g., CTB, rAAV2-retro, RV-ΔG—Hussein (2024) maps projections from the dorsal and median raphe nuclei to explore serotonergic networks. Their study clarifies the complexity and flexibility of serotonergic innervation across the brain, hence affecting areas from the arcuate nucleus (Arc) to the cortex. They strongly suggest that serotonergic circuits have different projection patterns and use both serotonergic and non-serotonergic pathways, suggesting complicated roles in controlling mood, arousal, and homeostatic activities (Ren et al. 2018; Hussein 2024).

From mapping to modulation, transcranial magnetic stimulation (TMS) is a powerful tool for circuit-specific intervention. Soleimani et al. (2025) propose that in patients with substance use disorders (SUDs), TMS might improve emotional control and decision-making by exciting the ventromedial prefrontal cortex (VMPFC)-amygdala circuit. Given that circuit disruption causes compromised emotional processing and impulsive drug-seeking behaviors, circuit-guided neuromodulation methods, such as recurrent TMS (rTMS), may offer therapeutic benefits (Hanlon et al. 2015; Soleimani et al. 2025).

While broader electrical neuromodulation techniques are advancing quickly, TMS is a particular type of focused circuit modulation. Balbinot et al. (2024) provides a thorough examination of how electrical stimulation affects neural substrates, including the direct triggering of action potentials and the alteration of synaptic plasticity and neurotransmitter release. They underline that tailoring therapeutic outcome depends on careful control of factors, including current intensity, pulse frequency, and electrode placement. These results highlight the need for circuit-specific knowledge in the development of neuromodulation treatments for numerous diseases, including movement disorders, depression, and chronic pain (Lozano and Lipsman 2013; Balbinot et al. 2024).

Finally, considerable anatomical and functional mapping research, such as that of Middleton and Strick (2000), will determine which circuits to target. Their seminal research on basal ganglia-thalamocortical and cerebellar-thalamocortical loops elucidated the interrelated activities of these regions in motor regulation and advanced cognitive processes. Several neuropsychiatric disorders, including PD and obsessive–compulsive disorder, have been linked to disruptions in these circuits (Middleton and Strick 2000; Habas et al. 2009). A logical therapeutic approach using the developing strong technology is based on a thorough knowledge of these complex networks.

Nanotechnology, photonics, viral tracking, and neuromodulation are changing our capacity to map, understand, and therapeutically control brain circuitry. Future efforts, including developmental timing, circuit-specific targeting, and network-level research, will be needed to convert these developments into feasible therapies for neurological and psychiatric disorders.

## Hippocampal Connectivity and Memory Processing

### Function of Hippocampal Circuits in Spatial Cognition and Memory Development

**Fig. 2:**
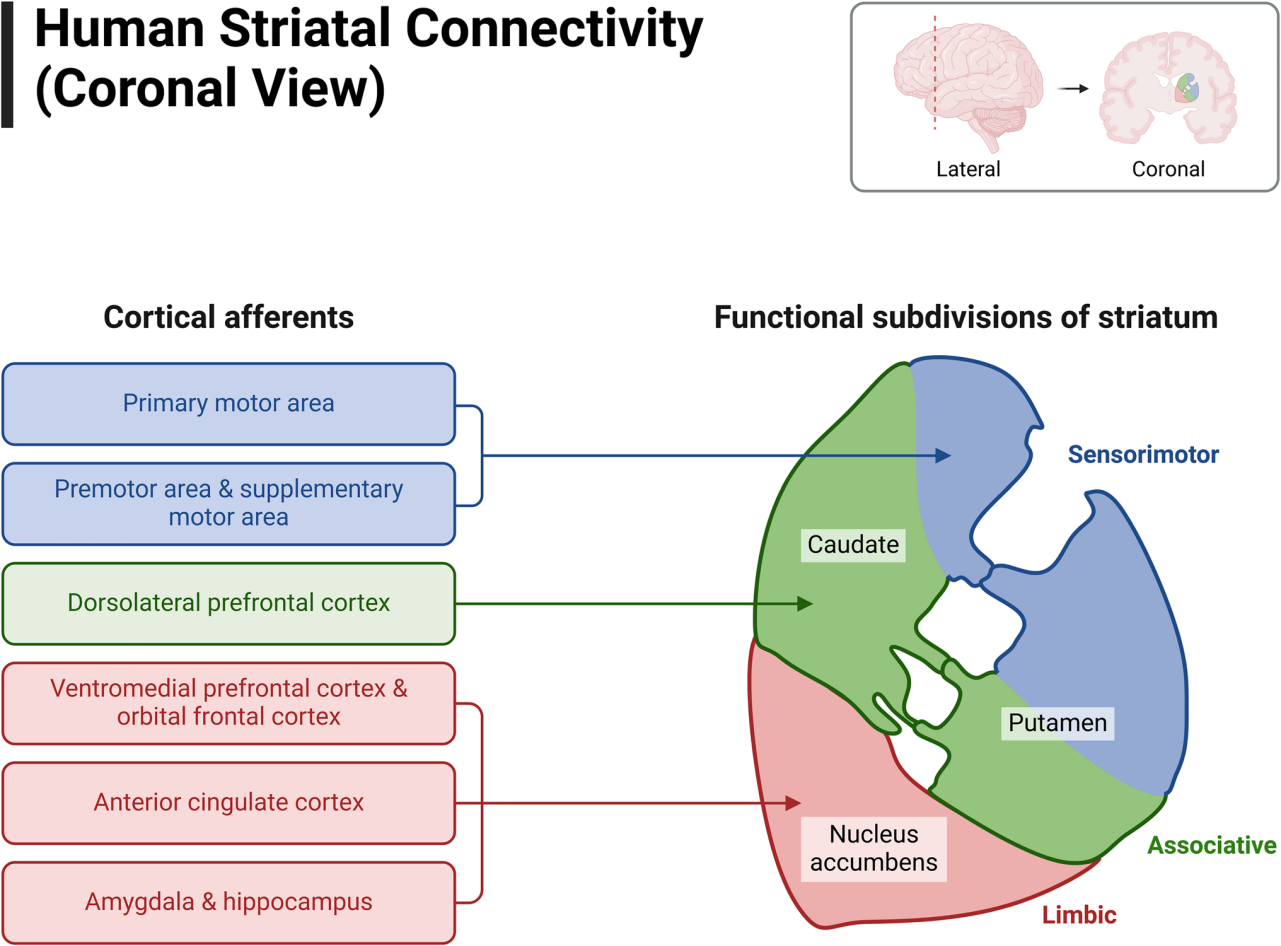
Human Striatal Connectivity (Coronal View). The graphic displays the cortical afferents and functional divisions of the human striatum in a coronal plane. The image shows several striatal areas—including the putamen, nucleus accumbens, and caudate nucleus—that get varying inputs from distinct cortical sites. Supporting the striatum’s involvement in reward processing, cognitive functions, and motor control, cortical afferents from the prefrontal cortex, motor cortex, somatosensory cortex, and other areas show specific striatal locations. The functional divide of the striatum is demonstrated: the associate striatum supports higher cognitive functions, the ventral striatum (nucleus accumbens) associated to reward and motivation, and the dorsolateral striatum primarily involved in motor control (Chuhma et al. 2017; McCutcheon et al. 2018)

Understanding how the brain adaptively changes behavior, stores memories, and maintains cognitive abilities throughout life requires mapping neuronal networks. The coronal view of human striatal connectivity shows how different parts of the cortex send signals to the striatum, like the putamen, caudate nucleus, and nucleus accumbens. These areas are involved in cognition, reward, and motor control (McCutcheon et al. 2018; Chuhma et al. 2017) (Fig. 2). Recent research emphasizes the significance of white and gray matter architecture and dynamic neuronal and epigenetic mechanisms in shaping brain network dynamics and supporting lifetime learning and adaptation.

**Fig. 3:**
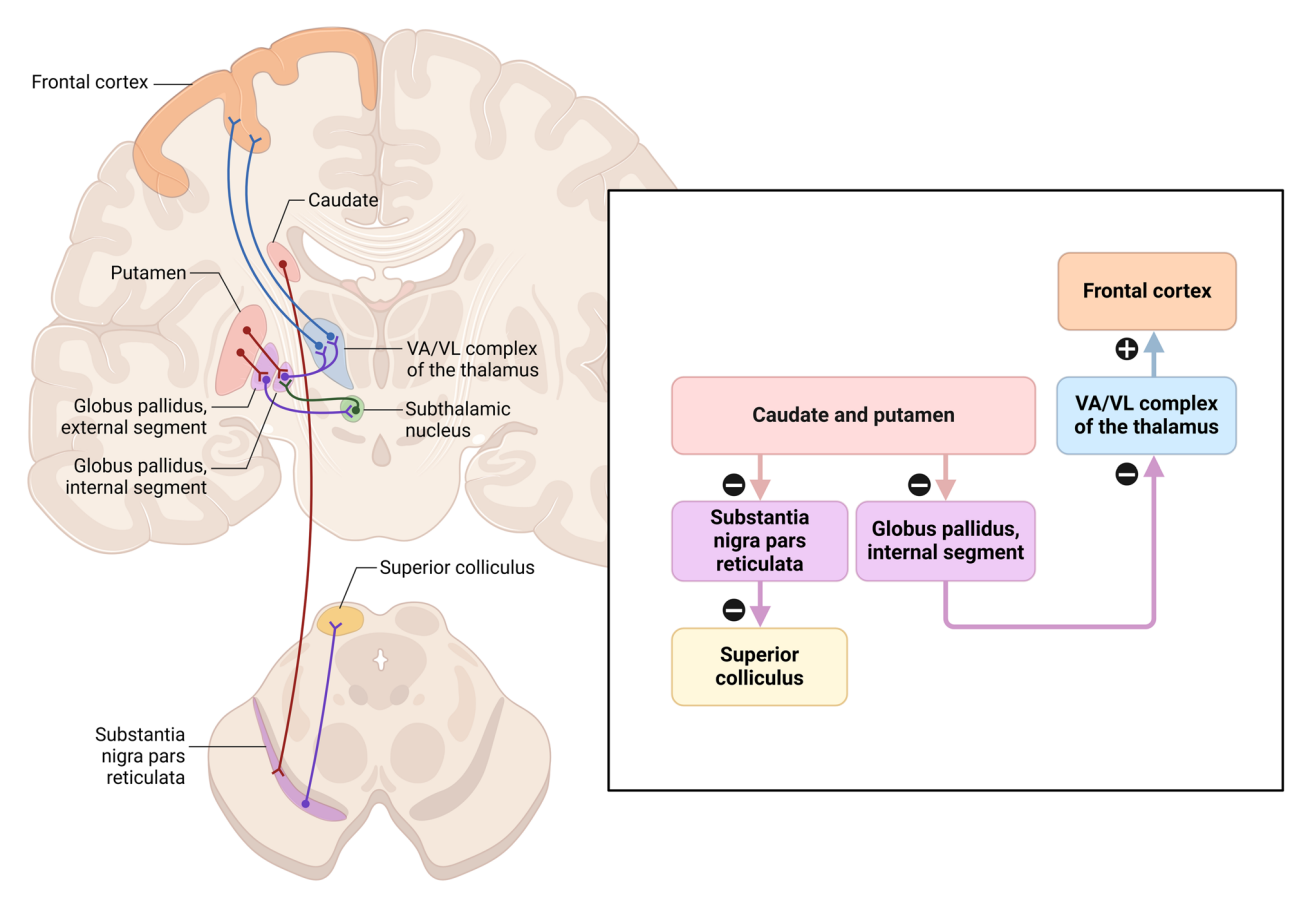
Basal Ganglia’s intrinsic circuitry and outputs. Emphasizing the complex neural network supporting cognitive and motor control, the schematic reveals the internal circuitry and outputs of the basal ganglia. The main components of the basal ganglia seen in the diagram are the striatum, globus pallidus (internal and external segments), substantia nigra (pars compacta and pars reticulata), and subthalamic nucleus. < It shows how the direct and indirect paths are reciprocally linked; the direct pathway promotes mobility while the indirect pathway forbids it. Along with the principal output channels from the basal ganglia—such as projections to the thalamus, which signals the cortex, and projections to the substantia nigra pars reticulata, which signals the brainstem motor areas—this figure also shows The interaction among these circuits determines the control of movement; PD and other neurological disorders are connected to dysfunction in this system (Lom 2018)

Li et al. (2023a, b) underlines the vital need for white matter integrity in maintaining large-scale brain network dynamics. Their study shows that the anatomical link provided by white matter tracts forms the basis for effective information transit between brain regions. Disruption of white matter pathways reduces network efficiency and is increasingly linked to cognitive deficiencies in aging and disease (Fields 2008; Li et al. 2023a, b). The basal ganglia circuitry highlights its internal components and output pathways, showing how the balance between the direct (movement-promoting) and indirect (movement-inhibiting) pathways regulates motor and cognitive control, with dysfunction linked to disorders like Parkinson’s disease (Lom 2018) (Fig. 3).

Examining the brain’s adaptive learning mechanisms, El-Gaby et al. (2024a) build on this network-based approach to particularly analyze the roles of hippocampal circuits and the medial frontal cortex in memory creation and behavioral generalization. Mice were conditioned on tasks with varying target locations, but the order of required activities was constant in their study. The mice showed a brain mechanism enabling quick generalization to unknown settings by displaying the ability to infer the task structure and properly carry out new variants from the first trial.

El-Gaby and colleagues identified specific neuronal clusters in the medial frontal cortex showing “goal-progress” tuning with firing patterns that dynamically mirrored the mice’s movement toward freshly set goals. Significantly, these neurons changed their activity to reflect different goal configurations without the requirement for re-learning, suggesting a kind of flexible, ordered memory buffering. Their results show that these dynamic brain patterns are consolidated during post-task sleep, stressing the importance of sleep in integrating new experiences into flexible behavioral frameworks (Euston et al. 2007; Gulati et al. 2017; El-Gaby et al. 2024a; Schwarzkopf et al. 2009).

Another paper points out the medial frontal cortex as a key hub for encoding, updating, and consolidating complex behavioral frameworks required for adaptive behavior. Dynamic, goal-oriented neural activity helps the brain transfer previously acquired patterns to new circumstances, a fundamental aspect of cognitive flexibility, as more and more research shows (Eichenbaum 2017).

At the same time, aging causes significant molecular and cellular changes that influence the anatomical and functional networks of the brain. Zemke et al. (2024a, b) thoroughly investigate age-related alterations in the human hippocampus by combining single-nucleus 3D genome organization, chromatin accessibility, DNA methylation, and transcriptomics from 40 adult specimens. Their study shows a notable decline in key non-neuronal cell types, especially astrocytes, especially those connected to synaptic support—oligodendrocyte progenitor cells (OPCs), and endothelial cells. At the same time, microglia showed a clear shift from homeostatic to a pro-inflammatory condition, suggesting a priming mechanism usually connected to neuroinflammation and cognitive decline (Hammond et al. 2019; Zemke et al. 2024a, b).

Zemke et al. document significant reprogramming of the three-dimensional genomic architecture in aged hippocampal cells, stressing the loss of enhancer-promoter links necessary for maintaining cell identity and synaptic function. This epigenetic loss points to a mechanism in which aging compromises cognitive resilience and cellular homeostasis. Notably, these three-dimensional genomic changes matched with significant DNA methylation alterations, linking structural chromatin remodeling to anomalies in gene expression seen in cognitive aging and neurodegeneration (Sun et al. 2023; Zemke et al. 2024a, b).

These results underline that maintaining brain adaptation over life depends on dynamic neuronal activity, structural integrity of white matter, and epigenetic flexibility working together. Any of these levels—genetic instability in hippocampal circuits, poor dynamic coding of behavioral goals, or white matter deterioration—can disrupt the brain’s ability to learn, adapt, and maintain cognitive processes. The studies of Li et al., El-Gaby et al., and Zemke et al. offer a thorough framework of neural circuit mapping tools and techniques, clarifying the intricacies of brain resilience and vulnerability.

### Important Results from Investigations into Connectedness in the CA1 and CA3 Areas

Recent developments in brain circuit mapping have improved our understanding of the hippocampus network structure and its relevance in spatial learning, memory, and age-related cognitive decline. Recent research clarifies particular noncanonical hippocampal pathways vital for cognitive functions by building on the results of Zemke et al. (2024a, b) and Lin et al. (2021) regarding age-related changes in hippocampal cellular composition and gene control. While Lin et al. (2021) underlined the relevance of unusual projections from the ventral CA1 (vCA1) to the dorsal CA3 (dCA3) region, Zemke et al. (2024a, b) investigated molecular and cellular changes linked with aging. Lin et al. challenged the conventional view of the hippocampus as controlled mainly by the canonical trisynaptic circuit (EC → DG → CA3 → CA1) by identifying notable inputs to dorsal CA3 from the subicular complex, perirhinal cortex (Prh), and vCA1 using retrograde viral tracing and chemogenetic suppression. Functional manipulation of the vCA1-dCA3 pathway revealed that while anxiety behaviors remained unchanged, disturbance of this link impeded object-related spatial learning and memory recall, suggesting that noncanonical hippocampal circuits have a unique and selective influence on cognitive activities (Lin et al. 2021).

Zhang et al. (2023a, b, c) built on this work, looking at similar circuits about AD using the 5xFAD mouse model. They showed that the loss of noncanonical hippocampal connections, particularly the vCA1-dCA3 circuit, led to problems in object-location memory. Behavioral abnormalities were linked to synaptic protein depletion and increased neuroinflammation, implying that the decline of spatial representation is an early factor in cognitive impairment in AD. These results draw attention to the relevance of unusual hippocampal circuits, both for normal spatial learning and memory and for clarifying how circuit-specific degeneration adds to pathology (Zhang et al. 2023a, b, c).

Apart from this study, Sun et al. (2019) investigated the subiculum-CA1 axis, a neglected pathway linked to spatial cognition. Using viral tracing and functional changes, Sun et al. defined a network whereby subicular neurons projecting to CA1 combine visual cortical inputs to improve object-place learning. The finding of a subiculum–CA1–perirhinal cortex feedback loop exposed a complicated mechanism linking spatial memory storage with sensory data. This circuit’s alteration compromised CA1 neuronal place fields and object-location memory, highlighting the importance of large hippocampus networks beyond the conventional circuit (Sun et al. 2019).

Sun et al. (2019) and Chen et al. (2023a, b) offer complementary views on hippocampal circuit function, stressing the vital need for synchronized neuronal activity patterns. Using in vivo calcium imaging, Chen et al. (2023a, b) showed that memory encoding is enabled by spatially and temporally coordinated activity inside CA1 microdomains. Neuronal ensembles relevant to behavior showed different spatial clustering, suggesting the hippocampus organizes memory traces into functionally specialized subregions. Disruption of these activity patterns might cause cognitive deficits and offer a mechanical connection between cellular activity and behavioral outcomes (Chen et al. 2023a, b).

By exposing noncanonical excitatory synaptic inputs from CA1 and the subicular complex directly targeting GABAergic interneurons in the CA3 area, Lin et al. (2023) clarified hippocampus microcircuitry even further. Their findings found a complicated feedback system in which excitatory modulation of inhibition affects CA3 network dynamics, sustaining circuit integrity during memory encoding and retrieval. This result challenges the traditional view of unidirectional hippocampal flow and emphasizes the importance of inhibitory circuit integration in preventing hyperexcitability and memory instability—ideas particularly relevant to conditions like epilepsy and AD (Dudek and Sutula 2007; Lin et al. 2023).

Building on the idea of inhibitory control, Grieco et al. (2023a, b, c) investigated a separate population of interneurons distributed across hippocampal and prefrontal cortical circuits co-expressing parvalbumin (PV) and cholecystokinin (CCK). Maintaining the excitatory/inhibitory balance within local circuits depends on the PV-CCK interneurons, which are defined by their quick firing patterns and sensitivity to endocannabinoid neuromodulation. Indicating a larger relevance for understanding neuropsychiatric disorders through the lens of microcircuit stability, the impairment of PV-CCK interneuron function has been linked to cognitive deficits and anxiety-related behaviors in schizophrenia and autism spectrum disorder models (Cardin 2018; Grieco et al. 2023a, b, c).

Patricia E. Sharp (2025) provides a critical historical and theoretical analysis tracing the evolution of hippocampal research from associative learning frameworks to the contemporary “cognitive map” theory. Sharp underlines that our understanding of the hippocampus as a dynamic and flexible network for encoding relational knowledge about space, objects, and events has been transformed by significant changes during the cognitive revolution and the rise of parallel distributed processing (PDP) models in addition to improvements in circuit mapping, imaging, and computational modeling. Emphasizing the necessary interaction between empirical evidence and evolving theoretical conceptions, her story shows the field’s movement toward increasingly complex models of hippocampal function (O’Keefe and Nadel 1978; Sharp 2025).

The above findings indicate that hippocampal function is supported by a complex network of canonical and noncanonical circuits, dynamic inhibitory control mechanisms, and spatially structured neuronal ensembles. Unraveling these complex networks has depended on innovations in viral tracking, chemogenetics, optogenetics, and high-resolution imaging technologies. Future work merging circuit mapping approaches with transcriptomic, epigenetic, and functional research will be important for understanding how hippocampal networks adapt—and fail—across age, neurodegeneration, and psychiatric disease.

## Neuroplasticity and Circuit Remodeling

### Functional Modifications in Cortical Plasticity

Neuroplasticity is the brain’s remarkable ability to change, remodel, and restructure itself in response to various stimulus, traumas, or environmental changes. Vital for learning, memory, and healing from neurological injuries, this dynamic process comprises structural and functional changes at the synaptic, cellular, and network levels. Circuit remodeling—encompassing alterations in synaptic strength, dendritic and axonal structure, and the balance between inhibitory and excitatory signaling—is at the heart of neuroplasticity. These modifications improve neuronal performance and are affected by mechanisms including glial cell connections, activity-dependent gene expression, and neuromodulatory signaling.

Understanding neuroplasticity provides vital knowledge on the cognitive flexibility of the brain, its potential to recover after neurodegenerative diseases, mental disorders, and brain injuries, and how these processes are compromised in such situations. This part looks at present research that has improved our knowledge of neuroplasticity, circuit reconfiguration, their consequences for cerebral function, and therapeutic treatments for disorders.

Parkins et al. (2024) provide important new perspectives on the spatial patterns of changes in brain activity across sensory areas in adult mice following visual deprivation. Their study used in vivo calcium imaging to track neuronal activity in the visual, auditory, and somatosensory cortices before and after the start of visual deprivation. They said that visual deprivation changed cellular activity in the primary visual cortex (V1) and faraway sensory areas, indicating cross-modal plasticity. Apart from a complex network reconfiguration of sensory processing-related networks, the changes included increased and decreased neuronal firing rates. These changes’ form and magnitude differ among sensory modalities, suggesting that the brain’s reaction to sensory loss is modality specific. This suggests that by changing activity in related sensory areas, the brain compensates for the lack of sensory input by restructuring itself. These results draw attention to the brain’s capacity for cross-modal plasticity, allowing functional changes in one sensory area when another modality is absent. This work improves our knowledge of neuroplasticity and offers potential therapeutic strategies to reduce the consequences of sensory deprivation and promote brain growth (Parkins et al. 2024).

Apart from cross-modal plasticity, recent studies have examined how neuroplasticity is affected by outside stressors, including radiation. Studies on cranial irradiation show that higher radiation doses, such as nine Gy, lead to a significant drop in synapse density, increased neuroinflammation, and a decline in cognitive performance. The observed effects were linked to lower brain-derived neurotrophic factor (BDNF) levels in the hippocampus and reduced expression of the neural plasticity marker cFos, indicating defective neuroplasticity (El-Khatib et al. 2024). RZ, a neuroprotective drug, appears to reduce these effects in subsequent treatment. RZ treatment taken orally over several weeks following radiation exposure not only restored synaptic density and cFos expression but also raised BDNF levels in the hippocampus, suggesting that RZ might be a therapeutic tool to enhance neuroplasticity and cognitive recovery in brain cancer survivors suffering receptor-induced cell death (RICD). Behavioral tests showed improvements in learning, memory, and memory consolidation in RZ-treated mice compared to vehicle-treated controls, supporting their neuroprotective qualities. This emphasizes RZ’s possible role as a translational neuroprotective mechanism helping to prevent or reverse cognitive deterioration following radiation treatment (El-Khatib et al. 2024).

Focusing on how sensory experiences, especially visual input, shape cortical development and neuronal function, Xie et al. (2024) used spatial transcriptomics to investigate the molecular mechanisms of neuroplasticity. Focusing on layer 2/3 (L2/3) glutamatergic neurons in the primary visual cortex (V1), their study found unusual gene expression patterns connected to different neuronal cell types and moods. Their results showed that visual experience changes two main gene expression programs: controlling neuronal cell types and monitoring cell statuses. Notably, visual deprivation changed the state of L2/3 neurons regardless of cell type-specific gene expression changes. The study also found a transcriptional continuity among several L2/3 cell types defined by a notable drop in B-type and C-type cells and an increase in A-type cells following visual loss. These changes in the neural structure of V1 are linked to V1 neural structure alterations, highlighting the importance of visual inputs in affecting neuronal activity and brain circuitry. Emphasizing the need of early sensory input to develop proper cellular variety and functional structure of the neocortex, the study provides important new perspectives on how sensory experiences affect the molecular and functional properties of neurons. Particularly in vital developmental periods, these results have significant significance for therapy interventions meant to offset the effects of sensory deprivation (Xie et al. 2024).

Recent studies have looked at how neuromodulatory signals affect neuroplasticity. Grieco et al. (2023a, b, c) investigated a specific subset of inhibitory interneurons—the parvalbumin (PV)-cholecystokinin (CCK) co-expressing cells, which are crucial for controlling the balance between excitatory and inhibitory impulses in circuits of the hippocampus and prefrontal cortex. By adjusting synaptic activity and preventing excessive neuronal activation, PV-CCK interneurons are crucial for maintaining network stability. Dysregulation of these cells has been linked to cognitive problems and anxiety-like behaviors, making them an interesting target for therapeutic interventions in neuropsychiatric disorders, including schizophrenia and autism. Grieco et al. (2023a, b, c) suggest that changing the function of PV-CCK interneurons could be a possible way to restore circuit integrity and reduce symptoms in those impacted. Their study emphasizes the complexity of inhibitory control in brain networks and its relevance for neuroplasticity and cognitive performance.

Ultimately, ongoing developments in neuroplasticity research and neural circuit mapping highlight the dynamic and flexible nature of the brain in reaction to sensory input, injury, and illness. The studies analyzed here, including those by Parkins et al. (2024), El-Khatib et al. (2024), Xie et al. (2024), and Grieco et al. (2023a, b, c), improve understanding of neural circuit reorganization and adaptation to various obstacles, therefore providing important insights into possible therapeutic strategies for increasing neuroplasticity and cognitive rehabilitation in neurological diseases and post-brain injury.

### The Function of Neurogenesis and Inhibitory Interneurons in the Adaptation of Brain Circuits

The adaptation of brain circuits is strongly affected by the balance between excitatory and inhibitory (E/I) synaptic activity, which is crucial for sustaining functional plasticity. Regulating synaptic activity, which affects network oscillations and sensory processing improvement, is dependent on inhibitory interneurons—especially those expressing parvalbumin (PV) and somatostatin (SST) (Koh et al. 2016). These interneurons control the cortical activity’s temporal and spatial dynamics, allowing adaptive changes in neural networks. Recent research highlights the importance of inhibitory interneurons and adult neurogenesis in reconfiguring brain circuits, with notable consequences for cognitive functions like learning and memory and neurodegenerative diseases (Aimone et al. 2014).

Neurogenesis in the adult hippocampus is a key process enabling circuit reconfiguration by adding freshly generated neurons into pre-existing networks. Learning, memory, and cognitive flexibility are improved by this method (Zhao et al. 2008). Moreover, neurodegenerative diseases, brain damage, and environmental factors could change the course of these processes. By improving plasticity and allowing circuit remodeling in response to damage or disease, therapy targeting the interaction between inhibitory interneurons and neurogenesis may offer new ways for treating psychiatric and neurological disorders (Barbier et al. 2016).

By looking at the transfer of embryonic inhibitory interneurons to restore juvenile-like cortical plasticity in the adult mouse visual cortex, Zheng et al. (2021) significantly advanced understanding of neural circuit reconfiguration. They particularly focused on PV interneurons, vital for controlling cortical excitability and plasticity. Transplanting these PV interneurons let the adult visual cortex reawaken cortical plasticity, hence enabling the brain’s adaptation to new sensory inputs similar to that of a juvenile brain. The Neuregulin (NRG1)/ErbB4 signaling pathway, recognized as a key mediator in this process, helped to include transplanted PV interneurons into host circuitry. By altering inhibitory interneurons and signaling pathways like NRG1/ErbB4, this work emphasizes the therapeutic promise of increasing cortical plasticity in the adult brain, hence offering hope for the recovery of brain function in age-related or injury-induced deficits.

Still, fully explaining the relationship between brain activity and complex behaviors remains difficult. According to Ozkirli et al. (2025a, b), reductionist approaches often overlook the complexity of brain-behavior connections. Though computer modeling for understanding neural systems has improved, the non-linear dynamics and high complexity of brain interactions create significant challenges in linking complex phenomena with single neuronal circuits. Their results emphasize that behaviors are emergent properties of complex neural interactions, and that the complexity of these interactions makes it difficult, if not impossible, to draw clear cause-and-effect connections between certain brain activities and behaviors. This calls for rethinking reductionist theories and a shift to more integrative strategies that can more properly handle the complexity of brain-behavior interactions (Ozkirli et al. 2025a, b).

Apart from the studies on interneuron transplantation, later ones have produced greater knowledge of plasticity, cerebral function, and the possibilities for creative therapies for neurological disorders. Examining the development of tonotopic and somatotopic maps inside the brainstem’s somatosensory system, Lee et al. (2024a, b) showed how sensory map organization was affected by peripheral receptor configurations. These sensory maps, vital for understanding vibrational signals from the environment, seem to increase the brain’s ability to distinguish spatial and frequency information. These findings improve our understanding of brain development and sensory processing.

Uchigashima and Mikuni (2024) developed “single-cell symptom mapping,” a technique aimed at mapping individual synapses at the molecular level, based on the comprehension of sensory systems. This method clarifies the role of synaptic connections in the flexibility and reconfiguration of brain circuits, hence allowing researchers to explore synaptic diversity and its relevance in neural computing. Elucidating the mechanisms that control brain plasticity, especially during development and in response to outside stimuli, requires understanding synaptic diversity at the single-cell level (Uchigashima and Mikuni 2024).

By increasing synaptic connections inside the thalamocortical circuit, essential for sensory and motor processing, Luo et al. (2025a, b) showed that physical activity, especially treadmill exercise, can have neuroprotective qualities. Their study showed that exercise increased excitatory synaptic input from pyramidal cells in primary motor cortex (M1) to posterior thalamic nucleus (Po), supporting the notion that physical activity could promote synaptic plasticity and neuronal resilience. The results highlight the need for brain-derived neurotrophic factor (BDNF) and TrkB signaling in enabling synaptic plasticity and stress resistance, suggesting novel treatment options for anxiety and other stress-related disorders (Luo et al. 2025a, b).

Geng et al. (2024) examined how indigenous and transplanted stem cells interacted to promote neurogenesis following ischemic stroke. Transplantation of exogenous stem cells increased the growth and development of native neural stem cells, hence promoting tissue regeneration and functional recovery. This study demonstrates the prospect of merging stem cell therapy with endogenous neurogenesis to increase results in neurodegenerative illnesses and cerebral injuries (Geng et al. 2024).

Shi et al. (2025a, b) looked at how cortical interneurons affect addiction and substance use disorders (SUDs). The evolution and durability of addictive behaviors depend on the variety of interneurons in the medial prefrontal cortex (mPFC) and their unique connection patterns. Their study shows that aiming at specific groups of cortical interneurons could offer a feasible therapeutic strategy for addiction, hence generating fresh knowledge of the physiology of addiction and potential treatments (Shi et al. 2025a, b).

Recent developments in neural circuit mapping technology and studies on neuroplasticity, stem cell transplantation, and exercise highlight the brain’s dynamic adaptability. Understanding the functions of inhibitory interneurons and neurogenesis in circuit reconfiguration better helps us develop new therapeutic ideas for neurological diseases, including neurodegenerative diseases, addiction, and brain trauma. Improving these techniques and tackling the complex problems related to brain-behavior mapping will call for more research.

## Neuromodulation Techniques for Neurotherapy

### Inhibition of Responses and Neural Networks

Recent developments in neuromodulation technology provide hopeful treatment possibilities for many neurological and behavioral disorders. These developments have improved our understanding of the neural circuitry controlling brain activity and highlighted the potential of using these technologies to treat many mental health diseases. The study of neural networks linked to response inhibition is critical since it is vital for processes of self-control and decision-making. Developing neuromodulatory treatments meant for diseases characterized by poor inhibitory control, including attention deficit hyperactivity disorder (ADHD), impulse control issues, and many neuropsychiatric disorders, requires an understanding of the brain’s response inhibition networks.

Focusing on the prefrontal cortex and insula, two important regions involved in controlling impulsive behavior, Osada et al. (2024a, b) conducted a thorough study of the brain circuits linked to response inhibition. Using sophisticated neuroimaging techniques such as functional magnetic resonance imaging (fMRI) and transcranial magnetic stimulation (TMS), the researchers identified the brain areas involved in suppressing prepotent responses and clarified the neural circuits supporting self-control. From perception to execution, the study showed that response inhibition triggers particular insular-prefrontal networks at various stages. The results show that the prefrontal cortex and insula interact dynamically, each performing a different function in different stages of the inhibition process. This thorough knowledge of the neural networks linked to response inhibition would significantly influence the development of focused neuromodulatory medicines. Activating certain brain areas with TMS or other neuromodulation methods, for example, might improve inhibitory control and operate as a therapeutic intervention for people with ADHD or other disorders marked by weakened self-regulation (Osada et al. 2024a, b).

Recent developments in neuromodulation technologies, including deep brain stimulation (DBS) and transcranial direct current stimulation (tDCS), are increasing treatment options for conditions linked with compromised neural circuits. For example, while tDCS has shown promise in altering cortical excitability and improving cognitive performance in people with depression and other mood disorders, DBS has successfully treated movement disorders like PD by concentrating on specific areas of the basal ganglia. These approaches offer non-invasive or slightly invasive ways to affect brain activity and restore neuronal circuits damaged by injury, disease, or developmental problems.

Furthermore, optogenetics—a novel technique allowing exact control of brain activity by light—has dramatically improved our knowledge of the neural networks linked to complicated behaviors. Optogenetics has been used to study response inhibition in animal models, clarifying the causal links between brain networks and inhibitory control (Warden et al. 2024). Careful activation or suppression of specific neurons allows researchers to track the real-time effects on behavior, hence offering a level of control and accuracy that was previously unattainable with more traditional methods. This has opened doors for therapeutic approaches directly targeting brain circuits linked to cognitive and behavioral issues.

Apart from neuromodulation’s potential therapeutic applications, current research has underlined the need for circuit-specific treatments in understanding and treating psychiatric diseases. Sadeh et al. (2024) found that improving specific brain circuits with focused neuromodulation could increase cognitive flexibility and reduce anxiety and depression symptoms. Their results suggest that tailored neuromodulatory treatments, designed to the particular brain circuits linked to every patient’s condition, might offer a more effective substitute for traditional uniform therapy.

Recent developments in neuromodulation technologies have produced new knowledge on brain networks linked to response inhibition and other complex behaviors. Neuroimaging, optogenetics, and neuromodulatory methods have helped scientists map the brain areas and networks controlling inhibitory control, hence opening new paths for therapeutic interventions. Neuromodulation is anticipated to become a more important tool in treating mental and neurological disorders as our understanding of these circuits develops, hence offering customized and focused therapy to improve patient outcomes.

### Neuromodulation in Mental Health Treatment

The role of glucagon-like peptide-1 (GLP-1) receptor agonists in mental health therapy has drawn significant interest because of their putative therapeutic advantages outside their usual use in the control of type 2 diabetes and obesity. Recent studies have highlighted the possible benefits of GLP-1 receptor agonists in treating many mental health disorders, including neurocognitive issues, depression, and substance addiction. The rising prevalence of neurodegenerative diseases like AD and other psychiatric illnesses that still challenge traditional therapies amplifies the relevance of these consequences.

Chiefly responsible for the control of glucose metabolism and insulin generation, GLP-1 is a peptide hormone. Besides their metabolic functions, recent research suggests GLP-1 and its receptor greatly affect brain activity and mental wellness. Emphasizing their ability to reduce the frequency of substance misuse, psychotic diseases, and seizures, Au et al. (2023) undertook comprehensive research on the neuropsychiatric benefits of GLP-1 receptor agonists. Their study underlined the possible neuroprotective and antidepressant qualities of these medications, which appear to be linked to their ability to control neuroinflammation, improve synaptic plasticity, and promote neurogenesis in important brain areas connected to mood control and cognition (Au et al. 2023, 2024).

Among the most fascinating aspects of GLP-1 receptor agonists in mental health is their neuroprotective qualities, particularly in neurodegenerative diseases like AD. Recent studies show that GLP-1 receptor activation could reduce the pathogenic processes linked to AD, including amyloid plaque formation, tau tangles, and synaptic dysfunction (Bäckman et al. 2023). According to preclinical studies, GLP-1 receptor agonists may increase cognitive function, reduce neuronal loss, and improve behavioral symptoms in animal models of AD, thus providing a hopeful, creative approach for treating AD (Sweeney et al. 2023). Human clinical studies have shown that GLP-1 receptor agonists may slow down disease progression in those with moderate cognitive impairment (MCI) or early-stage AD and have cognitive benefits (Li et al. 2023a, b).

GLP-1 receptor agonists also show antidepressant-like effects as they alter important brain regions linked to mood control, including the hippocampus and prefrontal cortex. Studies have indicated that in areas usually damaged in people with depression, GLP-1 receptor activation can enhance neuroplasticity and promote neuronal survival (Liu et al. 2023). The results suggest that, especially for those unresponsive to traditional antidepressant drugs, GLP-1 receptor agonists may be a viable therapy option for depression.

Apart from their apparent effects on cognitive and mood control, GLP-1 receptor agonists have been connected to reduced drug-seeking behavior, hence suggesting them as a potential therapy option for handling substance use disorders. Research shows that stimulation of GLP-1 receptors might influence the reward circuits of the brain, which are usually compromised in people with addiction. By changing the action of vital neurotransmitters, including dopamine, GLP-1 receptor agonists could reduce cravings and prevent relapses in people with substance use disorders (Zhang et al. 2023a, b, c).

These results encourage more research on the development of GLP-1 receptor agonists as treatment choices for several neurodegenerative and mental diseases. The neuroprotective and antidepressant effects shown in preclinical and clinical studies highlight the possibility of these drugs to provide new, unusual approaches for mental health therapy. Research still indicates that GLP-1 receptor agonists might be essential in managing addiction, neurological diseases like AD, and depression, so offering fresh possibilities for those with limited choices in traditional treatments.

### Aromatherapy and Sleep Illnesses

Initially used in aromatherapy for its calming and sleep-promoting qualities, lavender essential oil (LEO) has recently attracted much attention for its neuronal pathways that support improved sleep quality. Modern research has started identifying the specific brain areas and pathways involved in LEO’s therapeutic effects, offering a deeper understanding of how this natural medicine could treat sleep problems and related conditions. Using polysomnographic recordings to evaluate changes in brain activity during sleep, Ren et al. (2023) recently looked at how LEO affected sleep patterns in freely roaming C57BL/6J mice.

The study found that breathing LEO during the light (inactive) phase significantly lowered the latency to start non-rapid eye movement (NREM) sleep, extended the total duration of NREM sleep, and increased cortical electroencephalographic (EEG) slow-wave activity, a marker suggestive of restorative sleep. Two compounds known for their sedative qualities, linalool and d-limonene, were found to be the main components of LEO that appeared to affect these outcomes. The researchers also found that the GABAergic system in the central amygdala, a brain region crucial for emotional control and stress responses, helped promote LEO’s sleep-inducing effects.

Pharmacogenetic reduction of GABAergic neurons in the central amygdala, or disruption of the olfactory system, completely neutralized LEO’s effects on sleep, emphasizing the vital relevance of these systems in enabling its therapeutic efficacy. The central amygdala regulates sleep, anxiety, and stress responses, which is therefore quite important (Zhao et al. 2022). The participation of the olfactory system emphasizes the complex interaction between sensory input and the brain’s internal processes controlling physiological activities like sleep (Chen et al. 2023a, b). These results confirm the efficacy of lavender essential oil in treating sleep problems and provide a neurological basis for its historical use in conventional medicine.

This work enables the study of how specific brain circuits are modulated by aromatic compounds and how these circuits interact to influence behaviors, including sleep. Recent developments in chemogenetics and optogenetics have enabled more precise mapping of neural circuits linked to sleep control; future studies might apply these techniques to explore the particular pathways via which LEO exerts its effects. For example, the optogenetic activation or inhibition of certain central amygdala neurons or other sleep-related brain areas could clarify the precise role of GABAergic neurons in LEO’s sleep-promoting actions (Xie et al. 2023).

Upcoming studies could also use neuroimaging techniques such as functional magnetic resonance imaging (fMRI) and positron emission tomography (PET) to show changes in brain activity in reaction to LEO inhalation. Together with animal models, these methods would allow scientists to see the real-time activation of certain brain networks, improving their knowledge of how LEO affects sleep-related circuits. Studies like this could clarify the mechanisms by which sensory cues, such as smells, influence complex physiological states like sleep and wakefulness by linking brain circuit dynamics to behavioral results.

LEO’s therapeutic possibilities for sleep disorders could perhaps include various neurological conditions characterized by disrupted sleep, including anxiety, depression, and neurodegenerative illnesses. Studies done before show that those with worry and sadness often have sleep anomalies, and restoring normal sleep patterns might be a possible therapy path for these conditions (Li et al. 2023a, b). Sleep’s neuroprotective qualities, including the promotion of neuroplasticity and the removal of neurotoxic chemicals like beta-amyloid, may provide indirect benefits for conditions like AD (Wilson et al. 2022). Therefore, whether used alone or as part of a multi-modal treatment approach, LEO may have great relevance in the management of neurological diseases.

Taken together, modern neurobiological research increasingly supports the relevance of lavender essential oil in improving sleep quality, especially by means of its interaction with the GABAergic system and olfactory routes. Ren et al. (2023) provide a strong framework for understanding the neurological foundations of LEO’s sleep-inducing effects, hence enabling more research on the related brain circuits. Improved knowledge of the molecular mechanisms involved and developments in neural circuit mapping technologies might help LEO to be used therapeutically for several mood and sleep problems.

### Neuromodulation for Treatment of Autism and Post-traumatic Stress Disorder (PTSD)

Manocchio et al. (2023) look at how well neuromodulation methods—particularly transcranial direct current stimulation (tDCS) and repetitive transcranial magnetic stimulation (rTMS)—treat post-traumatic stress disorder (PTSD). Often linked with dysregulated brain circuits, particularly those in charge of anxiety and stress processing, PTSD is a serious condition. Promising therapeutic intervention is offered by neuromodulation treatments such as tDCS and rTMS, which can affect these circuits.

The study by Manocchio et al. emphasizes how various stimulation parameters—target hemisphere, stimulation frequency, and duration—affect the effectiveness of these therapies. Because of its fundamental involvement in executive functions, including emotional control, decision-making, and cognitive control, which are compromised in PTSD, the dorsolateral prefrontal cortex (dlPFC) is a primary target for neuromodulation therapy. The findings underline that by enhancing emotional control and lowering the hyperactivity of fear-processing circuits in the amygdala, targeting the dlPFC with different neuromodulatory strategies could help to lower symptoms.

Manocchio et al. (2023) underlines the importance of more research to improve stimulation techniques and acquire a better knowledge of the underlying neuromodulatory mechanisms, even if these studies show encouraging results. Improving the therapeutic efficacy of these methods will depend on optimizing stimulation parameters, including finding the most efficient stimulation frequency and the precise spatial coordinates of brain targets. Moreover, customized treatment plans based on personal brain activity patterns could increase the precision and efficacy of neuromodulation therapy for PTSD. This approach could lead to tailored treatments that are more effective and in line with the unique brain profiles of those suffering from PTSD, hence improving treatment results and providing hope to those struggling with this disability.

In the field of neural circuit mapping, where advances in neuroimaging and functional connectivity studies improve understanding of the disturbances in brain networks linked with PTSD, the idea of individualized therapy is particularly relevant. Using techniques like resting-state fMRI and magnetoencephalography (MEG), researchers can map the functional links of the dlPFC and other regions involved in emotional control, hence enabling more precise neuromodulation targeting (Shin et al. 2022). This development in circuit mapping technology is enabling customized neuromodulation treatments to fit for the particular neurological impairment of every patient.

Emphasizing the effect of customized continuous theta-burst stimulation (cTBS) on the social abilities of young children, Xiao et al. (2023) investigate the use of transcranial magnetic stimulation (TMS) for autism spectrum disorder (ASD). Particularly for individuals with low verbal communication, their study highlights the effectiveness of cTBS as a non-invasive neuromodulatory technique able to enhance social communication skills in children with ASD. The researchers showed in a double-masked, randomized controlled trial that children undergoing tailored continuous theta-burst stimulation (cTBS) targeting the left dorsolateral prefrontal cortex (DLPFC) exhibited notable improvements in social communication skills and lower autism severity. Maintained across a three-month follow-up, the improvements suggested that cTBS might have lasting positive impacts on social interaction and language development in children with ASD.

Xiao et al. (2023) offers interesting research since it offers a fresh perspective on how neuromodulation could reduce basic ASD deficits, especially in social communication and language development. These neuromodulatory treatments mainly target the dlPFC, which is crucial for social cognition, executive function, and language processing. Researchers are deepening their knowledge of how customized stimulation regimens can change this brain area to promote social behavior in children with ASD. According to Xiao et al. (2023), more study is required to explore the long-term effects of cTBS and to increase knowledge of the underlying brain mechanisms enabling these changes.

Improving the knowledge of how neuromodulation influences brain function in ASD requires neural circuit mapping techniques. Functional imaging techniques like fMRI and diffusion tensor imaging (DTI) offer thorough mapping of the anatomical and functional links between the DLPFC and other brain regions involved in social cognition and communication (Liu et al. 2022). Using these methods, researchers could track changes in brain connectivity brought on by neuromodulation, so helping to identify the neural networks enabling improvements in social communication abilities. Furthermore, advanced neurostimulation techniques such as optogenetics and chemogenetics, which enable the precise control of neuronal activity at the level of individual neurons and circuits, could be used in conjunction with TMS to improve our understanding of the brain’s response to focused stimulation (Luo et al. 2023).

Ultimately, studies on PTSD and ASD underline the potential of neuromodulation as a treatment option by offering understanding of how non-invasive techniques such as tDCS, rTMS, and cTBS might change dysfunctional neural networks. Promising to directly target brain areas linked to emotional control and social awareness, these therapies provide a creative way to address the basic symptoms of these conditions. More study is needed to clarify the neurological mechanisms supporting treatment success, fine-tune stimulation settings, and identify optimal brain targets thereby improving the efficiency of neuromodulation therapy. These projects will depend on advances in neural circuit mapping technology like neuroimaging and advanced stimulation techniques, which will enable more targeted and effective treatments for patients with PTSD, ASD, and other neuropsychiatric disorders.

### Cerebellar tDCS and Neural Connectivity

Using functional magnetic resonance imaging (fMRI), Maldonado et al. (2023) conducted a major investigation on how cerebellar transcranial direct current stimulation (tDCS) affected cerebello-cortical connection by means of this neuromodulation on brain networks. Their study found that cerebellar tDCS causes particular time-dependent changes in brain activity both right away and later following stimulation. When discussing specific neural circuits, the temporal component of brain modulation highlights the importance of stimulation time, hence stressing is a crucial element for improving neuromodulation treatments.

Through its broad connections with the cerebral cortex, the study showed that the cerebellum, usually recognized for its role in motor control, also greatly influences higher-order cognitive activities including emotional control and executive functioning. The findings showed that cerebellar tDCS changed the link between cerebellar and cortical regions, hence affecting brain networks linked to motor as well as non-motor activities. Notably, these changes occurred in a time-dependent manner, showing quick post-stimulation effects followed by delayed effects lasting several minutes to hours. The temporal fluctuations in brain network connectivity suggest that the timing of stimulation could be a key factor in the effectiveness of tDCS as a therapeutic tool. Tailoring stimulation regimens to the ideal temporal window could enhance treatment outcomes for individuals with mental and neurological disorders.

Maldonado et al. (2023)‘s study improves our understanding of how neuromodulation affects complex brain circuits and emphasizes the potential of cerebellar tDCS as a treatment option for several diseases, including motor disorders like PD and cognitive and psychiatric disorders like depression and schizophrenia. The cerebellum’s involvement in different brain operations, including emotional processing and cognitive control, suggests that targeting cerebellar networks could produce notable therapeutic effects.

These findings could be rather practically important in the management of neurological diseases including stroke and neurodegenerative ones. Studies on cerebellar-cortical networks in stroke patients show that the cerebellum is essential for motor recovery since it helps to reorganize brain circuits following injury (Miall et al. 2021). Using tDCS to affect cerebello-cortical connection might help doctors to promote better brain network remodeling, hence enhancing recovery results in people with neurological disorders.

This discovery affects the management of mental ailments including mood disorders and cognitive impairment. Recent studies show that the cerebellum helps control mood and cognitive control; problems in cerebellar-cortical connection have been connected to conditions such as major depressive disorder (MDD) and schizophrenia (Schmahmann 2021). Especially when traditional therapies show little efficacy, the ability to control cerebellar function by non-invasive techniques such as tDCS can open new possibilities for treating these conditions.

Apart from its therapeutic relevance, Maldonado et al. (2023) highlights the need for a more comprehensive knowledge of the neurophysiological mechanisms controlling the consequences of neuromodulation. Though the exact mechanisms underlying these changes are still unclear, their results suggest that cerebellar tDCS can cause changes in brain networks. Using advanced imaging techniques such as diffusion tensor imaging (DTI) and magnetoencephalography (MEG), future studies should explore the cellular and molecular mechanisms controlling the temporal effects of cerebellar tDCS to better define the structural and functional changes in brain connectivity (Tabei et al. 2022).

Personalized medicinal solutions may result from the combination of neuromodulation and brain circuit mapping technology. Formulating tailored neuromodulation protocols requires understanding the exact connection patterns of a patient’s brain given the very individualized character of brain networks. Resting-state fMRI and task-based fMRI are examples of functional connectivity studies that can identify the separate cerebello-cortical networks of individuals, hence enabling the tDCS treatment to target certain brain areas needing modulation (Brem et al. 2022).

Taken together, the work by Maldonado et al. (2023) represents a major development in the field of neuromodulation, clarifying how cerebellar tDCS affects cerebello-cortical connectivity and stressing the crucial relevance of timing in improving neuromodulatory approaches. These results are quite important for the development of treatment strategies for different psychological and neurological disorders. Research in this field will need to include advanced neural circuit mapping methods and tailored approaches to increase the efficacy and accuracy of neuromodulation treatment as it develops.

### Customized rTMS for Neural Plasticity

Emphasizing the context-dependent nature of its influence on brain networks, Ong and Tang (2025) conducted a thorough investigation of subthreshold repeated transcranial magnetic stimulation (rTMS) on neuronal plasticity. Their study showed that certain factors, including cortical layer, brain area, and stimulation settings, significantly affect the effectiveness of rTMS, which then affects gene expression linked to synaptic plasticity. These results offer crucial knowledge for improving rTMS as a therapeutic strategy for mental and neurological diseases by stressing the intricate interaction between stimulation protocols and promoting plastic changes in the brain.

Ong and Tang’s (2023) study underline the need of customizing rTMS protocols to the specific characteristics of individuals, including their cerebral structure, cortical excitability, and baseline neural activity. Tailoring rTMS to specific patient profiles can enhance the potential of the brain to adapt and reorganize in reaction to therapeutic treatments. The personalization of rTMS shows notable promise for improving treatment results for several conditions, including stroke, depression, and neurodegenerative diseases, where brain plasticity is often affected (Kim et al. 2022).

The researchers found that the particular brain area treated determines the effectiveness of rTMS. Stimulating the prefrontal cortex (PFC), for instance, is connected to improvements in mood control and cognitive processes; stimulation of areas like the motor cortex can promote motor recovery following brain loss (López-Alonso et al. 2020). The degree of plastic changes is significantly influenced by the specific cortical layers involved in the stimulation process. Studies show that whereas deeper layers (4–6) could need stronger rTMS to produce notable effects on synaptic plasticity, surface layers (layers 1–3) show more sensitivity to rTMS (Yap et al. 2021).

Moreover, the stimulation technique—whether low-frequency or high-frequency rTMS—affects plasticity in different ways. High frequency rTMS usually increases excitability and long-term potentiation (LTP), both of which are vital for synapse strengthening and the enhancement of memory and learning. On the other hand, low-frequency rTMS may cause long-term depression (LTD), which is required to suppress maladaptive neural circuits and restore homeostasis in situations including as pain or anxiety disorders (Huang et al. 2022a, b). A precise approach for controlling brain circuits in a context-sensitive and individualized way is provided by the combination of many stimulation frequencies.

Ong and Tang’s (2023) results show that rTMS’s temporal dimensions have a major impact on the outcomes. Since stimulation can either improve or impair task performance depending on its temporal window, the time of rTMS administration in connection to a certain cognitive or motor task may influence its effectiveness (Jahanshahi et al. 2020). Moreover, the interaction between rTMS and other neuromodulatory therapies, such as pharmaceutical treatments or behavioral interventions, may modify or amplify its effect on brain plasticity (Pascual-Leone et al. 2021).

These findings underline the need for future studies to improve rTMS procedures by considering both the physiological characteristics of the brain and the specific clinical situation of the person. Examining the genetic and epigenetic underpinnings of plasticity in reaction to rTMS could provide important insights for finding biomarkers predicting which patients are most likely to gain from neuromodulatory treatments (Tavakkol et al. 2022). People with certain genetic variations influencing synaptic plasticity pathways can show a more noticeable reaction to rTMS, suggesting that customized approaches could significantly improve treatment outcome.

Ultimately, the study by Ong and Tang (2025) provides strong proof that rTMS, tailored to specific cerebral characteristics, can increase brain plasticity and improve therapeutic outcomes for various neurological and psychological conditions. But, as the field develops, more research is clearly required to refine rTMS techniques and grasp the fundamental mechanisms propelling its effects on the brain. Modern neural circuit mapping tools—including real-time fMRI and optogenetics—allowing researchers to better understand how rTMS affects brain networks at the level of individual neurons and synapses. More precise and effective neuromodulatory treatments will result from this improved knowledge, hence giving fresh hope for those suffering from different neurological disorders.

### Vagus Nerve Stimulation and Cholinergic Networks

Capone et al. (2023) examined how transcutaneous auricular vagus nerve stimulation (taVNS) affected human cholinergic brain networks, especially with regard to short-latency afferent inhibition (SAI). Quantifying the suppressive impact of sensory inputs on motor cortex excitability, SAI is a known method for evaluating cholinergic neurotransmission. Contrary to findings from animal models showing a considerable effect on cholinergic transmission with comparable stimulation, Capone and colleagues found that taVNS had no appreciable effect on SAI (Pereira et al. 2020). This difference between human and animal results suggests possible fundamental variations in the operation of the cholinergic system between species, particularly with respect to the modulation of cortical networks by vagus nerve stimulation. Emphasizing the need for more thorough research to clarify the processes by which taVNS influences cholinergic systems in people, the paper highlights the complexity of translating preclinical findings into human therapies.

This finding calls for a careful study of the particular ways taVNS affects brain circuits linked to cognitive, emotional, and sensory processing. Though animal models provide important insights, human brains could react differently because of structural and functional variations. Humans and animal models may differ in their distribution of cholinergic receptors and vagus nerve neuronal projection configuration (López et al. 2021). Improving taVNS methods to optimize therapeutic outcomes in individuals will depend on understanding these differences, particularly in relation to disorders like AD, depression, and PTSD, where cholinergic dysfunction is implicated (Ghosh et al. 2022).

Apart from taVNS, several other non-invasive neuromodulation techniques, including transcranial magnetic stimulation (TMS), transcranial direct current stimulation (tDCS), and continuous theta-burst stimulation (cTBS), show great promise for treating neurological and psychosocial diseases. These methods have attracted great attention for their ability to affect brain activity and improve outcomes in conditions like PTSD, depression, ASD, and sleep problems. Showing effectiveness in reducing depression symptoms and improving cognitive function, transcranial magnetic stimulation (TMS) has been used to change activity in the dorsolateral prefrontal cortex (dlPFC), an area linked with mood control and cognitive control (Huang et al. 2022a, b). With therapeutic consequences including motor rehabilitation and improvements in social communication skills in individuals with ASD, tDCS and cTBS have been shown to influence cortical excitability and plasticity (López-Alonso et al. 2021).

More precise targeting and evaluation of neuromodulation effects have been made possible by recent developments in brain circuit mapping technologies such as functional magnetic resonance imaging (fMRI) and electroencephalography (EEG). For example, fMRI lets researchers look at how different stimulation protocols affect brain network connection in real time, hence revealing the mechanics behind certain neuromodulatory strategies. Moreover, EEG can capture the quick electrophysiological effects of neuromodulation, hence providing more information on changes in brain oscillations and synchronization (Miller et al. 2020). These technologies are advancing neuromodulation approaches by giving a more thorough grasp of interregional brain communication and the impact of stimulation on this interaction.

Research on non-invasive neuromodulation techniques is becoming clear that customized approaches are absolutely vital to maximize their effectiveness. Setting stimulation protocols requires careful consideration of parameters like a person’s neuroanatomy, baseline brain activity, and the particular features of the ailment being treated (Gershon et al. 2021). Furthermore, developments in computer modeling and machine learning techniques are enabling more precise forecasts of the influence of different stimulation patterns on brain circuits in individual patients, therefore enabling more tailored and successful therapies (Thielscher et al. 2022).

Their effective use in clinical practice depends on continuous study to improve neuromodulation methods. Our ability to create more effective, tailored treatments for many neurological and psychosocial diseases will increase as we gain knowledge of the basic neural routes. Future studies should focus on the long-term effects since the sustainability of the benefits of neuromodulation is still under active investigation.

Taken together, the results of Capone et al. (2023), Capone et al. (2025)) emphasize the complexity of translating preclinical findings into human applications and the possibility of neuromodulation to affect brain activity and improve therapeutic effects. Realizing the complete potential of neuromodulation in clinical settings will depend on continuous advances in brain circuit mapping technologies and tailored treatment approaches.

## Neuronal Circuit Function in Neurological Diseases

### Neural Network Changes and Hippocampal Dysfunction in AD

Recent studies have provided important new understanding of how AD disrupts brain networks, particularly those linked to spatial memory and cognitive function. Elucidating the pathophysiological changes in AD has mostly centered on the hippocampus, a vital brain region for recording and retrieving spatial and episodic memories. Using the triple-transgenic AD (3xTg-AD) mouse model, known for the accumulation of amyloid plaques and tau neurofibrillary tangles, Lin et al. (2022a, b) explored the spatial coding capacity of hippocampus neuronal ensembles. Though there was an increase in calcium activity inside CA1 neurons, their study found a notable decline in spatial representation in 3xTg-AD mice. Paradoxically, the higher brain activity did not match improved spatial memory or navigation. Scores of spatial information fell and aged animals in particular lost location specificity. Hippocampal neurons’ firing patterns grew sparse, which caused a notable loss of spatial coherence. The results show that hyperactivity in AD does not enhance cognitive performance; rather, it exacerbates cognitive deficiencies, possibly caused by a dysregulated hippocampal network insufficiently supporting spatial memory storage (Lin et al. 2022a, b).

Changes in the excitation–inhibition (E-I) balance across the brain highlight even more the idea that changed hippocampal dynamics in AD cause cognitive deficits. Li et al. (2025a, b) investigated the evolution of the excitatory-inhibitory balance in AD using resting-state functional MRI (fMRI) combined with a Multiscale Neural Model Inversion (MNMI) framework. From cognitively normal people to those with moderate cognitive impairment (MCI) and AD, their study found a progressive drop in this balance. In crucial brain areas, including the hippocampus and anterior cingulate cortex, the disruption was more pronounced; inhibitory connections suffered more than excitatory ones. The authors suggest that the difference between excitatory and inhibitory neurons might significantly affect the cognitive deficits observed in AD, including those in memory, learning, and general cognitive flexibility. These results highlight the need to restore E-I balance as a possible treatment goal in AD (Li et al. 2025a, b).

Brain circuit mapping technologies, including contemporary neuroimaging techniques, have become vital tools in recent years for clarifying the complex neuronal changes in AD. Both animal models and human individuals provide important insights on brain network connectivity and neuronal ensemble activity using functional MRI (fMRI) and calcium imaging. Resting-state brain networks have been shown to be somewhat effective in fMRI as have monitoring of E-I balance disturbance in AD (Li et al. 2025a, b). Animal models have also used multi-photon microscopy and optogenetics to observe real-time neuronal activity with high spatial resolution, hence enabling studies to investigate the impact of amyloid plaques and tau tangles on synaptic activity and network connections (Shah et al. 2023). These approaches offer a more complex understanding of the processes by which AD damages brain function at the level of single neurons and synaptic connections.

Recent studies on the dynamics of neuronal firing patterns in AD have used electrophysiological methods including in vivo electroencephalography (EEG) and local field potential (LFP) recordings. These methods provide a direct evaluation of the oscillatory activity inside neural circuits, which is vital for understanding the dysregulation of brain networks in AD. AD people and animal models have been reported to have lower theta and gamma band oscillations, which are required for hippocampal-dependent cognitive activities including memory and learning (Sanchez et al. 2024). The spatial and memory deficits typical of the disease are probably caused by oscillatory synchronization disturbance in important brain areas.

Optogenetic and chemogenetic technologies are also being used more often to regulate particular brain circuits in AD models to test theories about their involvement in cognitive deficits. These methods allow for the investigation of causal connections between network collapse and cognitive deficiencies by means of exact control over brain activity. Studies show that optogenetic therapy to restore hippocampal network coherence can partially alleviate memory difficulties in AD mice (Cai et al. 2023). Such studies offer vital knowledge on future treatment strategies meant to restore normal brain function in AD.

In conclusion, research on how AD affects brain networks, especially in the hippocampus and other memory-related regions is still being done. Lin et al. (2022a, b) and Li et al. (2025a, b) provide compelling proof that cognitive decline in AD results from hippocampal hyperactivity and disrupted excitatory-inhibitory balance. The inclusion of advanced neural circuit mapping technologies, including fMRI, calcium imaging, and electrophysiological techniques, is producing a more complete knowledge of the influence of the disease on brain networks. These technologies not only enhance our understanding of the basic mechanics of AD but also provide possibilities for finding novel treatment targets and treatments to reduce cognitive loss in those with AD.

### Basal Ganglia Circuitry: Cognitive-Motor Dysfunction

**Fig. 4:**
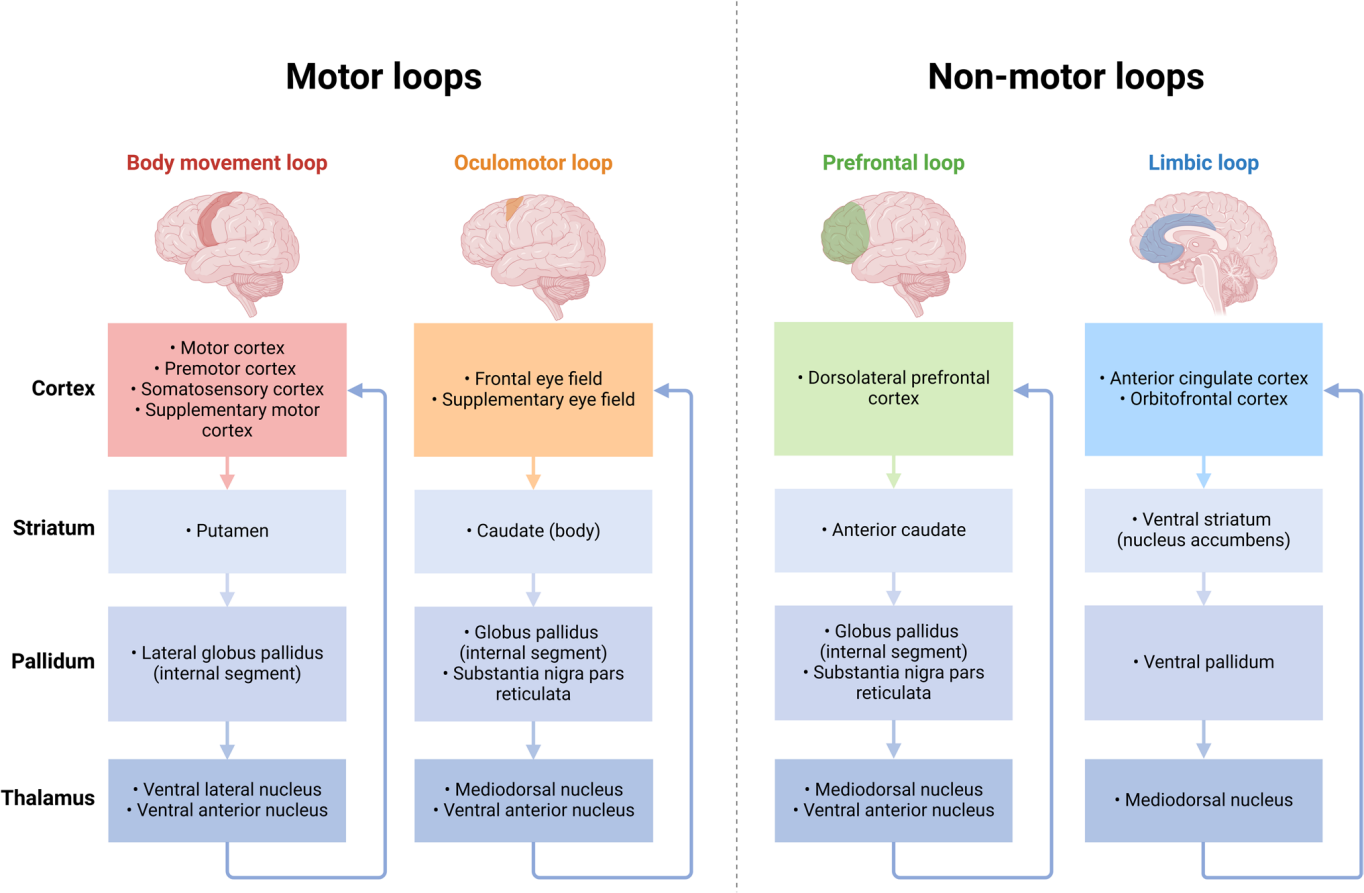
Basal Ganglia Motor and Non-motor Loops. The structure of the motor and non-motor loops of the basal ganglia is described here, with particular attention to their striatal targets, pallidal outputs, cortical inputs, and thalamic projections. The motor loops, including the oculomotor and body movement loops, process inputs from areas like the frontal eye field, somatosensory cortex, and motor cortex. These signals are transmitted through the putamen or caudate nucleus and project via the globus pallidus and substantia nigra to thalamic nuclei involved in the regulation of motor and eye movements. The non-motor loops handle Cognitive and emotional processing, including the limbic and prefrontal loops. The anterior caudate and thalamus receive signals from the dorsolateral prefrontal cortex, which are then integrated via the prefrontal loop. After processing inputs from the orbitofrontal and anterior cingulate cortex, the limbic loop projects to the nucleus accumbens and, eventually, the mediodorsal thalamus. These circuits collectively demonstrate how important the basal ganglia are for coordinating emotional, cognitive, and motor processes (Fineman 2020)

Apart from hippocampal issues, deficits in other crucial areas, especially those linked to cognition and motor control, notably help to cause neurological and neurodegenerative diseases. Vounatsos and Gittis (2025a, b) investigated projections from the exterior part of the globus pallidus (GPe) to the retrorubral field (RRF), showing intricate circuits comprising both dopaminergic and GABAergic neurons. Their results showed that by changing excitatory and inhibitory outputs, the GPe controls RRF neuronal activity, affecting downstream motor and cognitive activities. These results highlight the complex integrative role of the basal ganglia in maintaining coordinated motor execution and cognitive flexibility (Fig. 4).

Once considered a motor control center, the basal ganglia are now increasingly recognized for their vital roles in higher-order cognitive tasks, including learning, executive function, and decision-making (Redgrave et al. 2010; Lanciego et al. 2012). Classic movement disorders like PD have been linked to disturbances in basal ganglia circuits, especially those connected to imbalances between dopaminergic excitation and GABAergic inhibition. They are also more and more connected to the cognitive decline seen in AD (Kordower et al. 2001; Apaydin et al. 2002). Vounatsos and Gittis’s (2025a, b) work builds on this understanding by showing that disturbance in GPe-RRF projections could directly cause the motor and cognitive problems seen in AD.

Recent circuit mapping and neuroimaging studies back this theory by showing that basal ganglia-thalamocortical circuits are damaged in the early stages of AD, which corresponds with deficits in executive function, attention, and motor coordination (Grothe et al. 2016; Barbosa et al. 2020). Both clinical AD postmortem samples and experimental AD models have revealed degeneration of GABAergic interneurons and a drop in dopaminergic transmission within these circuits (Rinne et al. 1998; Limon et al. 2012).

The combined influence of dopaminergic and GABAergic pathways in the basal ganglia highlights the therapeutic promise of altering these systems. Novel techniques to reduce cognitive and physical deterioration in AD could come from pharmacological policies targeting dopamine receptors, GABAergic signaling, or their regulatory networks. Furthermore, advanced technologies such as optogenetics and chemogenetics allow the precise modification of particular basal ganglia circuits, hence offering experimental confirmation for circuit-targeted treatment (Kravitz et al. 2010; Witten et al. 2010).

Understanding the complex function of basal ganglia dysfunction in AD provides a strong basis for creating creative therapy ideas aiming to restore balance inside these fundamental neural networks and improve cognitive and motor outcomes.

### Genetic Mutations and Neuroinflammation: Their Roles in AD

Genetic risk factors significantly influence the progression and variation of AD besides disruptions in brain networks. One element connected to increased risk and severity of AD pathology is the TREM2^R47H mutation. Renowned for its significant amyloid pathology, the 5xFAD mouse model was investigated by Johnston et al. (2024a, b) using Multiplexed Error-Robust Fluorescence In Situ Hybridization (MERFISH) to assess the TREM2^R47H mutation’s influence. Their results showed that, regardless of amyloid plaque presence, the TREM2^R47H mutation increased synthesis of brain-derived neurotrophic factor (BDNF) and its receptor Ntrk2 in cortical excitatory neurons. This suggests that TREM2 mutations can naturally change neuronal physiology before the start of significant amyloid build-up.

Johnston et al. (2024a, b) also observed notable changes in the mix of glial and neuronal populations associated with both mutation and amyloid illness. The TREM2^R47H mutation selectively changed BDNF signaling pathways and increased inflammatory responses of glial cells, including microglia and astrocytes. By upsetting neurotrophic support and immunological signaling, these changes probably aggravate neurodegeneration. This work emphasizes the link between genetic risk variations and cellular network abnormalities in AD, suggesting that genetic factors may affect disease progression by changing glial reactivity and directly affecting neuronal circuitry and plasticity.

The results of this work fit broader trends showing that TREM2 and related immunological pathways are key neuroinflammatory mediators in AD (Deczkowska et al. 2018; Ulland and Colonna 2018). TREM2 mutations such as R47H impede microglial response to amyloid plaques, causing poor plaque compaction, altered phagocytosis, and heightened neuroinflammation that more disturbs synaptic networks (Jay et al. 2017). Furthermore, single-cell transcriptome studies show that microglia with TREM2 mutations exhibit a disease-associated microglia (DAM) phenotype linked with amyloid and tau disorders (Keren-Shaul et al. 2017).

These results underline the need for cell type and region-specific mapping methods to clarify how genetic changes damage brain circuitry with great resolution. Across several brain regions, technologies such as MERFISH, spatial transcriptomics, and single-cell multi-omics provide unmatched insights into the reactions of different cell populations to genetic and pathologic pressures (Zhang et al. 2023a, b, c). The geographical and cellular resolution is crucial as immunological and neurodegenerative responses might vary greatly depending on the local environment, regional susceptibility, and the specific subgroup of affected cells.

Including genetic risk assessment into neural circuit mapping projects could enable precision medicine approaches in AD. Tailored therapies targeting the TREM2 pathway or the alteration of BDNF signaling may reduce neuroinflammation and synaptic dysfunction, hence slowing disease progression. Recently, Studies using antisense oligonucleotides to control TREM2 expression have shown promise in preclinical animals (Zhao et al. 2023).

Johnston et al. (2024a, b) underline that future therapeutic developments for AD must consider the complex, connected roles of genetic variation, neural circuit dysfunction, and immunological dysregulation. Creating customized treatments to affect the cellular and molecular pathways driving dementia needs multi-modal, cell-type-specific mapping tools.

### Early AD: Amyloid-β Accumulation and Memory Affections

Elucidating the pathogenesis of AD requires understanding the consequences of amyloid-β (Aβ) deposition on neuronal circuits and cognitive function, especially with early memory and executive skill deficits. Recent studies have emphasized the basal frontotemporal cortex, an area linked to temporal and episodic memory processing.

Vanderlip et al. (2024a, b) looked examined the relationship between Aβ build-up in the basal frontotemporal cortex and temporal memory categorization deficiencies, the cognitive ability to distinguish between temporally nearby yet different memories. Their results strongly link increased Aβ build-up and lower performance on temporal event discrimination tests. Early Aβ pathology therefore suggests a significant loss of the temporal organization of memories, a vital component of episodic memory and cognitive flexibility. Notably, these changes show before the clinical onset of global cognitive impairment, stressing the basal frontotemporal brain as a possible early treatment target.

The results of Vanderlip et al. (2024a, b) match current knowledge showing that Aβ accumulation varies across the brain and that its regional distribution could predict domain-specific cognitive problems (Palmqvist et al. 2017). During the early phases of disease progression, the basal forebrain and frontotemporal areas show more sensitivity as Aβ-induced synaptic damage causes circuit disconnection and reduced plasticity (Bero et al. 2012; Busche and Hyman 2020).

Advanced brain circuit mapping tools produce ever more thorough insights into these processes. Resting-state functional MRI (rsfMRI), diffusion tensor imaging (DTI), and connectomics-based modeling, among other techniques, have shown that Aβ-related pathology particularly affects long-range connectivity in memory and executive networks, including the default mode network (DMN) and frontotemporal circuits (Jacobs et al. 2018). Aβ build-up in these areas is linked to hyperconnectivity and network fragmentation before clear atrophy, suggesting that functional dysregulation comes before structural decline.

By linking molecular changes—including the downregulation of synaptic genes and inflammatory alterations—with localized Aβ accumulation and network dysfunction, spatial transcriptomics and multiplexed imaging techniques such as MERFISH and seqFISH + enhance our understanding (Chen et al. 2023a, b). These high-resolution spatial tools let scientists see how amyloid illness changes glial and neuronal cells throughout different brain regions.

The cognitive problems related to Aβ accumulation in the basal frontotemporal cortex, as shown by Vanderlip et al. (2024a, b), highlight the need for early, spatially focused treatment. Interventions aimed at reducing Aβ or improving synaptic resilience in vulnerable regions, including the basal frontotemporal cortex, could help to maintain temporal memory abilities and delay significant cognitive decline. Emerging therapeutic approaches using targeted ultrasound, localized immunotherapies, or AAV-delivered anti-Aβ biologics are studying how spatially precise targeting may improve treatment efficacy and prevent off-target consequences (Leinenga et al. 2021; Chen et al. 2024a, b).

Ultimately, improved neural circuit mapping technologies and precise molecular profiling are clarifying how Aβ pathology disturbs brain networks in the early stage of AD. Particularly for episodic memory and temporal discrimination, this all-encompassing approach promises to produce early diagnostic tools and region-specific treatments meant to reduce first cognitive symptoms.

### Linking Therapeutic Insight and Neural Change in AD

An in-depth grasp of the changes in brain circuits associated with AD derives from evaluating the synergistic impacts of genetic mutations, hippocampal impairment, and basal ganglia circuit anomalies. The disruption of neural network dynamics, especially the delicate balance between excitatory and inhibitory transmission, vital for maintaining cognitive activities including memory, learning, decision-making, and spatial navigation is the fundamental cause of these pathological changes.

Growing evidence points to amyloid build-up (Aβ) as a cause of synaptic toxicity as well as a promoter of significant circuit disturbance across linked brain areas (Busche and Hyman 2020). Genetic predispositions, such as mutations in the APP, PSEN1, and PSEN2 genes, exacerbate the vulnerability of brain networks by upsetting synaptic homeostasis and accelerating amyloid accumulation (Selkoe and Hardy 2016; Lin et al. 2022a, b). Disruptions in hippocampus circuitry, particularly in excitatory pyramidal neurons and inhibitory interneurons, greatly impede memory encoding and retrieval processes, hence producing early cognitive symptoms of AD (Li et al. 2025a, b).

Apart from the hippocampus, recent research has revealed that basal ganglia circuits also undergo significant pathological alteration in AD. Vounatsos and Gittis (2025a, b) clarified intricate projections from the exterior section of the globus pallidus (GPe) to the retrorubral field (RRF), suggesting that GPe neurons control both dopaminergic and GABAergic outputs. This complex interplay emphasizes the role of the basal ganglia in cognitive control as well as physical regulation. These circuit failures could help explain the cognitive decline and movement problems sometimes observed in AD patients. The simultaneous disturbance of dopaminergic and GABAergic pathways in the basal ganglia suggests possible therapeutic targets for restoring circuit homeostasis (Vounatsos and Gittis 2025a, b).

Recent studies using circuit mapping techniques including optogenetics, chemogenetics, and advanced viral tracing have clarified the impact of amyloid pathology on functional connectivity. Hyperexcitability, synaptic failure, and reduced network synchronization follow from disturbances in long-range excitatory projections and aberrant local inhibitory signaling (Johnston et al. 2024a, b; Vanderlip et al. 2024a, b). Observed in the hippocampus, cortex, and subcortical regions at the start of disease progression, this pathological hyperactivity suggests network instability precedes significant neuronal loss.

These results highlight the absolute need for drugs that are both circuit-specific and stage-specific. There is great promise in early treatments meant to restore the natural balance of excitation and inhibition, protect synaptic integrity, and normalize aberrant neural networks. Future therapeutic strategies might include gene therapy to address particular molecular and circuit-level issues as well as targeted pharmacological treatments and neuromodulatory interventions.

Eventually, the development of precision treatments meant to block or reverse the cognitive and motor disabilities typical of AD will be guided by a more complete knowledge of how genetic, molecular, and circuit-level dysfunctions interact to compromise brain function.

## Cognitive Deficit and Schizophrenia

### Early THC Exposure: Effects on Opioid Sensitivity

Recent developments in neural circuit mapping technologies have significantly improved our understanding of how early-life experiences affect brain development and later vulnerability to neuropsychiatric disorders. Hubbard et al. (2024) in Neuropsychopharmacology looked at how adolescent exposure to tetrahydrocannabinol (THC), the main psychoactive component of cannabis, affected opioid reactions in adulthood. Their study suggested that early cannabis use can sensitize brain pathways, hence raising the likelihood of opioid addiction in later life as rats under THC during adolescence showed more sensitivity to morphine in maturity.

The focus of the findings was on changes in the reward circuitry of the brain, particularly in the mesolimbic dopamine system and the endogenous opioid system. Substantial rearrangement of synaptic architecture in important areas such as the nucleus accumbens, ventral tegmental area (VTA), and prefrontal cortex (PFC) was shown by neural circuit mapping using viral tracers, electrophysiological, and calcium imaging approaches. Rats given THC showed altered inhibitory GABAergic input and higher excitatory activation of dopaminergic neurons, which caused dysregulated dopaminergic signaling during opioid therapy (Hubbard et al. 2024).

The findings of the study fit an increasing body of research showing that adolescent cannabis exposure disrupts necessary stages of synaptic pruning and neural circuit development, therefore causing lasting changes in excitatory-inhibitory balance and reward sensitivity (Lubman et al. 2015; Volkow et al. 2016). Adolescence is a developmental stage of greater flexibility during which the brain’s reward and executive control systems are particularly vulnerable to outside influences. Exposure to exogenous cannabis during this time could lead to lasting consequences including altered opioid receptor expression, disturbed endocannabinoid signaling, and maladaptive circuit rearrangement (Rubino and Parolaro 2016; Renard et al. 2017).

Adolescent THC exposure also reduces the top-down regulating effect of the prefrontal cortex on subcortical reward regions, as indicated by neural circuit tracing and optogenetic studies, hence increasing the probability of drug use disorders (Cass et al. 2014). These circuit-level changes could result in increased opioid sensitivity as well as more broad deficits in decision-making, impulse control, and emotional regulation observed in those with a history of early cannabis use.

Hubbard et al. (2024)’s results highlight the need of preventive initiatives targeting adolescent cannabis use since it tends to alter brain circuitry, hence increasing susceptibility to addiction. Furthermore, they underline the crucial role of advanced brain circuit mapping technologies such as viral-genetic tools, in vivo imaging, and chemogenetics—in clarifying the cellular and synaptic circuits that underlie vulnerability to substance use.

Future studies should concentrate on determining the molecular mediators of these circuit changes, exploring reversibility using focused treatments, and assessing the translational relevance for human populations vulnerable to concurrent cannabis-opioid use issues.

### Cognitive Map in Schizophrenia

Recent developments in neural circuit mapping technologies have begun to clarify the notable influence of disruptions in spatial navigation and memory circuits on the pathophysiology of schizophrenia. Nour et al. (2024) undertook a major study looking at the role of cognitive maps—internal representations of spatial situations mostly driven by the hippocampus and entorhinal cortex—in schizophrenia. Their results show that abnormalities in the generation, maintenance, and use of these cognitive maps cause not only secondary symptoms but also core cognitive dysfunctions observed in schizophrenia.

Fundamentally based on functional hippocampal place cells, entorhinal grid cells, and their linkages to the prefrontal cortex and thalamus, cognitive maps (O’Keefe and Nadel 1978; Moser et al. 2008). High-resolution functional MRI (fMRI), diffusion tensor imaging (DTI), and calcium imaging in animal models have revealed abnormalities in these circuits in schizophrenia. Nour et al. (2024) particularly showed that changes in hippocampal-prefrontal connection damage spatial memory and flexible navigation, maybe clarifying the shortcomings in working memory, executive function, and reality monitoring usually seen in affected persons.

Recent circuit mapping studies using chemogenetic and optogenetic techniques show that genetic mutations connected to schizophrenia, especially those affecting NMDAR function, cause synaptic plasticity deficits in hippocampal CA1-CA3 circuits and the dorsolateral prefrontal cortex, therefore aggravating cognitive mapping deficits (Nakazawa et al. 2017; Datta et al. 2022). These shortcomings support Nour et al.’s claim that faulty cognitive mapping could be a basic feature of schizophrenia rather than a secondary consequence by causing spatial disorientation and more general cognitive disintegration.

According to the hippocampus-cortical disconnection theory of schizophrenia (Heckers and Konradi 2015), decreased hippocampal output activity leads to insufficient prefrontal control, hence impairing the development of consistent mental images of the surroundings. This concept supports the results of Nour et al. and emphasizes the need for early treatments targeting hippocampal-cortical circuitry.

Subsequent studies should continue to use advanced neural circuit mapping methods, such as connectomics, single-cell RNA sequencing combined with spatial transcriptomics, and in vivo multi-photon imaging, to elucidate the particular neuronal subtypes and synaptic mechanisms causing cognitive map disturbance. This knowledge could help create creative circuit-based treatments like targeted neuromodulation (e.g., deep brain stimulation or transcranial magnetic stimulation) and pharmaceutical strategies to restore the excitatory-inhibitory balance in prefrontal and hippocampal networks.

These results taken together underline the need for research on cognitive map dysfunction as a strong foundation for understanding the cognitive symptoms of schizophrenia and points of interest for the development of specialized treatments oriented on cognitive retraining.

### Functional Connectivity in Resting States: Major Depressive Disorder (MDD)

In their 2024 study described in Molecular Psychiatry, Hagen et al. evaluate resting-state functional connectivity in people diagnosed with major depressive disorder (MDD) using ultra-high field 7 Tesla functional MRI. The study of 31 Major Depressive Disorder (MDD) patients combined with 27 healthy controls matched for age and gender revealed noticeable patterns of hyperconnectivity and hypoconnectivity in some brain locations. Apart from variations in occipital interhemispheric connectivity, MDD patients had hyperconnectivity in thalamocortical pathways and hypoconnectivity in basal ganglia-cortical networks. These findings imply that perturbation in basal ganglia-thalamo-cortical circuits causes the fundamental pathophysiology of major depressive disorder, therefore affecting emotional processing and perception. Research emphasizes high-resolution fMRI as crucial in identifying minor changes in brain networks guiding MDD treatment (Hagen et al. 2024). Neuropsychiatric Disorders and Empathic Pain Crucially essential brain areas implicated in this process are the anterior insula, anterior cingulate cortex, and mirror neuron networks. Yang et al. (2024) on the brain pathways linked to empathetic pain, the capacity to share and understand another person’s suffering. Neuroimaging studies and mouse models help to clarify the cerebral foundations of sympathetic pain, therefore underlining the relevance of particular neuronal circuits and neurotransmitters. Many neuropsychiatric disorders, including psychosis, autism, and schizophrenia, demonstrate empathy deficits, so the creation of new therapeutic approaches depends on an awareness of these paths. More studies on empathetic pain would, the study underlines, elucidate empathy shortcomings in neuropsychiatric illnesses and support the creation of creative pain management techniques (Yang et al. 2024).

### Target Astrocytes for Epilepsy Treatment

Chen et al. stress their significance in regulating neuronal activity and preserving cerebral homeostasis, then consider how astrocytes might be used to treat epilepsy. Astrocyte failure aggravates epilepsy by throwing off the balance between excitatory and inhibitory impulses, thereby impairing metabolic homeostasis and so encouraging neuroinflammation. Defective astrocytes can disrupt synaptic plasticity and brain connections, generating hyperexcitability and seizures. The findings imply that focusing on astrocytes could treat epilepsy by restoring equilibrium in neuronal communication, lowering inflammation, and correcting defective brain circuits. This method is unlike traditional treatments that aim at reducing seizure symptoms (Chen et al. 2016).

### Control of Chronic Pain and Cognitive Ability

Brennecke et al. (2025) look at how various strategies of chronic pain treatment influence cognitive capacity. Their method addresses supplementary, physical, psychological, and medicinal therapies. The review notes that specific therapies, including cognitive-behavioral therapy (CBT), significantly increase cognitive performance, while others show little effect. More research is, the authors contend, needed to grasp the cognitive consequences of long-term opioids completely. They stress the importance of tailored treatment strategies considering the cognitive side effects of different therapies and advocate more excellent studies to investigate the processes behind these effects, thereby enhancing pain management and cognitive health (Brennecke et al. 2025).

### Glial Cell Damage in ASD: Autism Spectrum Disorder

Viewing the function glial cells—more notably, astrocytes and microglia—have in the pathophysiology of autism spectrum illness (ASD), Inamdar, Gurupadayya, and Sharma ask these cells to define preserving brain equilibrium, changing synaptic activity, and response to damage. The dysregulation of glial cells in autism spectrum disease can partly be explained by modified signaling pathways—particularly the mTOR pathway—and epigenetic modifications influencing gene expression. Active microglia and reactive astrocytes create an inflammatory milieu that aggravates synaptic defects and damages brain circuitry. The authors argue that targeting glial cell dysfunction with medications, including cell-based treatments, synaptic modulators, and anti-inflammatory pharmaceuticals, would effectively lower ASD symptoms. They propose innovative techniques, including stem cell therapy and gene editing, that could help restore normal glial function and offer new therapeutic prospects (Inamdar et al. 2025).

Díaz et al. (2024) investigate brain indications connected to therapeutic responses in anxiety- and post-traumatic stress disorder (PTSD) afflicted juvenile patients using neuroimaging as a predictive tool for therapy efficacy. Their research underlines the essential traits of these disease’s abnormalities in the amygdala-prefrontal circuitry, notably the anterior cingulate cortex (ACC) and ventromedial prefrontal cortex (vmPFC). Functional MRI related to improved emotional control following evidence-based treatments, including cognitive-behavioral therapy (CBT), showed reduced amygdala activation and enhanced vmPFC-amygdala connectivity. Changes in brain volume in areas including the ACC and hippocampus prediction of treatment outcome were shown by structural MRI. These findings encourage tailored treatment approaches since they show the opportunities of neuroimaging as a prediction tool for therapeutic success. The study supports the mix of neuromodulatory therapies, including transcranial magnetic stimulation (TMS), to boost therapy efficacy and improve long-term results for children and adolescents suffering from PTSD and anxiety (Díaz et al. 2024).

This various research has revealed both the possibilities for focused therapy and the role of the brain in mental health issues. Emphasizing the dynamic changes in neuroscience and psychiatry, this paper explores cannabis’s effects on glial cell regulation in autism, cognitive deficits in schizophrenia, and opioid sensitivity. To better grasp these pathways and translate these results into more efficient, tailored treatments for various neuropsychiatric and neurological diseases, future research will be vital.

### Interventions in Cognitive Error Generating Neural Networks

Zhang et al. (2025) underline the complicated function of neural networks in cognitive impairment, stressing that brain connection and function changes can lead to deficiencies in perception, attention, decision-making, language, reasoning, and memory. They consider several pathogenic elements that compromise neural circuit integrity and maintenance, compromising cognitive capacity. The authors stress developments in research tools such as chemical genetics, optogenetics, and brain imaging that have significantly improved our knowledge of neural circuit dynamics and their connection with cognitive functions. The study finishes by considering possible therapy strategies for brain networks and their fundamental role in maintaining cognitive capacity. The authors support the creation of fresh approaches to preserve or repair neural circuit integrity and propose that these concepts might enhance treatments for several cognitive diseases (Zhang et al. 2025).

### Effects of Immune Modulation on Brain Performance in Cerebral Palsy

Building on the general discussion on neural circuit anomalies, Kitase et al. (2025) look in a preclinical model of cerebral palsy (CP) the effects of CXCR2 immunomodulating medication on white matter integrity, motor performance, and cognitive findings. Acknowledged for their part in inflammatory responses, the alteration of the chemokine receptor CXCR2 has become a possible treatment strategy for neuroinflammatory illnesses. Using a model for cerebral palsy, newborn rats suffering hypoxic-ischemic injury were given a CXCR2 antagonist in their investigations. Although it did not lower the cognitive losses linked with the injury, their data reveal that CXCR2 inhibition maintained the microstructural integrity of white matter paths and enhanced motor function. According to the findings, CXCR2 immunomodulation might not aid in lowering the cognitive abnormalities connected with cerebral palsy, even if it may preserve white matter integrity and enhance motor performance. Based on Kitase et al. (2025), a more complete therapy of CP could rely on the mix of therapeutic approaches aimed at inflammation pathways and additional mechanisms generating cognitive abnormalities. Disease Pathology and Excitement-Inhibition Imbalance.

From immunological control in chronic pain to the effect of neurological imbalance on disease pathology, Hicks et al. (2025) probe the anatomical and functional linkages between the cerebellum and behavior over the lifetime. Their primary interests are how aging influences cerebellar integrity and its connection to motor and cognitive capacity. The study used advanced imaging techniques to reveal age-related alterations in cerebellar connectivity—including compensatory adaptations in specific regions and decreased connections in others. Particularly in motor and cognitive functioning areas, reduced performance on motor, memory, and attention tests was linked to decreases in cerebellar volume. These findings underline the important role of the cerebellum in preserving both motor control and many cognitive processes, therefore challenging the conventional wisdom on its sole responsibility for motor activities. This study clarifies the brain paths connected to aging-related cognitive and motor deficits (Hicks et al. 2025).

### PD and Circuit-Based Therapies

Recent developments in neural circuit mapping technology have created new paths for targeted therapeutic interventions in neurodegenerative illnesses including PD (PD). One encouraging strategy is altering certain brain circuits connected to motor impairment. Jackson et al. (2025a, b) looked at the therapeutic possibilities of changing the pallidothalamic circuit in a rat model of PD in a landmark work. Using sophisticated optogenetic technologies, scientists targeted the route linking the motor thalamus and the globus pallidus internus (GPi), a vital circuit for the control of voluntary motor control.

Their studies showed that optogenetic regulation of excitatory and inhibitory inputs to the motor thalamus efficiently restored more normal firing patterns, which had been substantially altered due to dopaminergic neuron degeneration—a hallmark of PD disease. Specifically, by selectively stimulating inhibitory pallidal afferents and balancing excitatory thalamocortical outputs, they may rectify abnormal oscillatory activities connected with bradykinesia, stiffness, and tremor. Treated rats therefore showed better mobility, less stiffness, and better motor coordination when compared to untreated PD models.

Significantly, this circuit-specific modulation strategy has benefits over more traditional therapies including systemic medication or deep brain stimulation (DBS). Although efficient, traditional DBS can occasionally result in off-target symptoms like speech problems, mood changes, or gait abnormalities owing to its rather broad stimulation area (Lozano et al. 2019). Likewise, systematic L-DOPA treatment can lead to long-term problems such as dyskinesias and reduced effectiveness (Obeso et al. 2017). In contrast, the approach of Jackson et al. offers a foundation for more exact, flexible, and possibly safer treatments that can fine-tune the activity of certain neuronal populations inside the motor circuit.

Furthermore, their research fits with new studies stressing the importance of circuit-level accuracy in managing movement disorders. Recent developments in chemogenetics—Designer Receptors Exclusively Activated by Designer Drugs, DREADDs—have also shown the possibility of precisely altering motor-related circuits to reduce PD symptoms (Kravitz et al. 2010; English and Venance 2021). Likewise, closed-loop DBS devices that change stimulation parameters depending on real-time brain feedback are now under clinical development to improve the selectivity and efficacy of neuromodulatory therapies (Herron et al. 2017).

According to Jackson et al. (2025), future studies should give circuit-specific pharmacological medicines or gene therapies meant to restore aberrant network activity within discrete motor circuits first priority. Such focused strategies could provide long-lasting symptom control, tailor therapy depending on individual circuit dysfunctions, and reduce side effects. Moreover, knowing the exact neuronal subpopulations and synaptic interactions most open to intervention will depend on including circuit mapping methods including optogenetics, calcium imaging, single-cell RNA sequencing, and connectomics (Gradinaru et al. 2010; Deisseroth 2021).

Jackson et al. (2025) results overall show a notable advance toward precise neuromodulation in PD, from symptom suppression to circuit reengineering. Eventually, this paradigm change could change the clinical management of PD and kindred neurodegenerative motor diseases.

#### Circuit Change in PD

Recent developments in neural circuit mapping have significantly improved our understanding of how microstructural abnormalities in key brain networks drive neurodegenerative diseases such as PD (PD). Wang et al. (2024a, b) conducted a thorough investigation of the cortico-striato-thalamo-cortical (CSTC) circuit, a fundamental network controlling motor and cognitive functioning, building on earlier studies on motor circuit dysfunctions. Using sophisticated neuroimaging techniques like diffusion tensor imaging (DTI) and connectomic analysis, the study uncovered notable microstructural alterations in the CSTC pathways of PD (PD) sufferers.

Notably in the motor cortex, striatum, thalamus, and prefrontal cortex, Wang et al. found notable changes in axonal integrity, synaptic connectivity, and white matter coherence in key CSTC loop nodes. These abnormalities showed a strong association with the level of motor symptoms—for example, bradykinesia, rigidity—and cognitive deficits—for example, executive dysfunction, reduced working memory—present in the patients. Various executive and cognitive problems were greatly influenced by degeneration in the dorsolateral prefrontal cortex (DLPFC) and its striatal connections, hence stressing the fundamental role of the CSTC circuit in both movement and higher-order cognitive regulation (Wang et al. 2024a, b).

Motor planning, habit formation, reward processing, and decision-making—processes greatly affected in PD—are all acknowledged to involve the CSTC circuit (Alexander et al. 1986; Haber 2016). Disruptions in this circuitry—stemming from dopaminergic neuron loss, α-synuclein pathology, or neuroinflammatory changes—are now recognized as a basic mechanism driving PD pathogenesis (Obeso et al. 2014). By mapping these structural changes at high resolution, Wang et al. offer fresh insights that could guide the creation of customized neuroprotective or circuit-restorative treatments.

This study underlines that in PD and other neurodegenerative diseases age-related network fragility, immunological dysregulation, and glial dysfunction might exacerbate neural circuit degeneration (Salter and Stevens 2017; Leng and Edison 2021). Studies coming out show that ongoing inflammatory signaling by active microglia and astrocytes helps CSTC circuits to degrade, hence compromising synaptic integrity (Ransohoff 2016).

These results underline the vital need to develop customized drugs that can either prevent circuit disintegration or promote remyelination and synaptic repair of affected pathways. Currently researched to restore CSTC circuit function are creative treatment plans including activity-dependent neuroprotective therapies, gene therapy, circuit-targeted chemogenetics, and regenerative techniques using glial reprogramming (Gradinaru et al. 2010; Luo et al. 2020).

The studies done by Wang et al. (2024a, b) and others taken together suggest that neurodegenerative diseases should be more often viewed as disorders of network dysfunction than only as conditions defined by regional brain atrophy or cellular death. Understanding the different patterns of circuit disruption linked to diseases like Parkinson’s allows researchers to more successfully create network-based treatments that target the fundamental reasons of both motor and cognitive impairments, therefore offering hope for more successful disease-modifying therapies.

## Possible Routes and Clinical Uses

### Methods for Restoring Circuits Derived from Stem Cells

Stem cells’ therapeutic use to restore brain circuitry has advanced noticeably. Wen et al. (2025) underline that donated stem cells have a complicated influence on the damaged microenvironment in spinal cord injury (SCI) outside simple neuronal regeneration. These cells secrete extracellular matrix components, neurotrophic factors, and cytokines that enhance the survival and function of endogenous neurons and also alter local immunological responses, guiding microglia and macrophage activity toward a pro-reparative phenotype. Wen et al. underline the ability of transplanted stem cells to create synaptic links with host neurons, hence enhancing motor and sensory recovery. This great impact shows that stem cells’ therapeutic potential in spinal cord damage goes beyond basic cellular replacement to make them vital controls of the regeneration environment (Wen et al. 2025).

Bipolar microsecond electric pulses (\u00b5sPEFs) were found to modulate induced neural stem cells (iNSCs) and mesenchymal stem cells (MSCs) by Innamorati et al. (2024). Though µsPEFs had small initial biological effects, significant increases in iNSC proliferation were observed within 72 h, suggesting that electrical stimulation could be used to boost stem cell-based regenerative therapies (Innamorati et al. 2024). This points to fresh prospects for bioelectric control to improve stem cell efficacy in medicinal uses.

Furthermore, Revah et al. (2022) showed that human cortical organoids can reach significant maturity, integrate into current host circuits, and create functional synaptic connections when transplanted into the mouse brain. Comprising organized layers, neuronal development, synapse formation, and electrophysiological activity in line with host networks, the transplanted organoids displayed fundamental traits of human cortex structure. These results support the use of cortical organoids as strong models for studying human neurodevelopment and as possible regenerative tools for rebuilding brain circuits in neurodegenerative diseases (Revah et al. 2022).

Recent research has underlined the need of scaffold biomaterials in conjunction with stem cells to enhance integration and linkage (Silva and Ferreira 2024), implying that combinatorial approaches could greatly enhance regeneration outcomes.

### Neuroinflammation and Epileptogenesis

Emphasizing the crucial functions of the prefrontal cortex, amygdala, and anterior cingulate cortex, Morawetz et al. (2024) provides a transdiagnostic viewpoint on emotional dysregulation in mental disorders. Malfunctions in these areas relate to conditions including anxiety, sadness, and borderline personality disorder. The study indicates that restoring balance in these circuits might improve emotional control in several disorders, hence supporting a shift to circuit-targeted therapy rather than diagnosis-specific approaches (Morawetz et al. 2024).

Simultaneous development has been made in circuit-specific epilepsy treatments as optogenetic and chemogenetic approaches to directly alter overactive circuits and reduce seizure activity (Bernstein et al. 2025).

### Neurodegenerative Diseases and Neurology

Chemogenetic techniques offer new possibilities for circuit analysis and therapeutic intervention in PD. Targeted stimulation of D1 dopamine receptor-expressing medium spiny neurons (D1-MSNs) by DREADDs in a non-human monkey model of PD, according to Chen et al. (2024a, b), led to improved motor function. Emphasizing the possibility to target certain basal ganglia circuits to restore motor and cognitive abilities, this precision neuromodulation technique (Chen et al. 2024a, b).

Novel methods such as single-cell connectomics and activity-dependent tagging, which provide unmatched resolution in defining defective circuits linked to neurodegenerative diseases, help to highlight the results (Zhang et al. 2023a, b, c).

### Neural Pathways, Immune System, and Autism

Particularly in relation to neurodevelopmental disorders, more attention is being paid to the role of the immune system in the formation and operation of brain circuits. Using induced pluripotent stem cells (iPSCs) derived from a bi-allelic NRXN1α deletion, Bose et al. (2025) investigated microglial dysfunction in autism spectrum disorder (ASD). These microglia, the researchers found, were lacking in enabling neural network growth and showed higher levels of the pro-inflammatory cytokine IL-6. Pharmacological reduction of IL-6 signaling greatly restored suitable network architecture, suggesting IL-6 as a key regulator of microglial-neuron connections throughout brain development (Bose et al. 2025). This study draws attention to the importance of neuroimmune interactions in ASD and offers immune-targeted therapeutic strategies.

Studies done concurrently point to the maternal immune activation model of ASD as further suggesting cytokine signaling disturbance in the lasting changes of brain circuits (Estes and McAllister 2024).

### Oncology and Neuroscience

The combination of neuroscience and oncology has produced new ideas for preserving cognitive function throughout cancer therapy. El-Khatib et al. (2024) found that in a mouse model, synaptic plasticity and cognitive function were preserved by brain-derived neurotrophic factors (BDNF) following cranial irradiation. Cranial radiation often causes cognitive decline from hippocampal damage; therefore, BDNF-based therapies could reduce these negative consequences and improve the quality of life for cancer sufferers (El-Khatib et al. 2024).

Other studies suggest that concentrating on neurogenesis-promoting pathways, including Wnt and Sonic Hedgehog (Shh) signaling, may further enhance neuroprotective effects after radiation (Antonelli et al. 2021).

### Psychiatric Neuromodulation

Techniques of neuromodulation—including transcranial magnetic stimulation (TMS)—are rapidly gaining prominence as adjunctive treatment in psychiatry. Findings from the NExT experiment by Conelea et al. (2024) on the effectiveness of TMS aimed targeting OCD-related circuits to enhance the outcomes of exposure and response prevention (ERP) treatment in adolescent OCD patients are presented. Aiming to control damaged frontostriatal circuits, this combined TMS-ERP approach could improve patient participation in behavioral therapy as well as symptom severity (Conelea et al. 2024).

Apart from OCD, creative TMS protocols are being researched for sadness, PTSD, and substance use disorders, showing promise for circuit-specific neuromodulation treatment (Cole et al. 2020).

### Using Stem Cells in Neural Regeneration

Yuan and Zhang (2024) investigate the intricacies of human neuronal transplantation into damaged or sick brain tissue. Successful integration depends on developmental synchronicity between transplanted neurons and host circuitry, favorable extracellular circumstances, and appropriate guiding cues. They caution that problems such as aberrant circuit formation, immunological rejection, and tumorigenicity have to be carefully handled. Advances in gene editing and nanomaterials, meantime, are making the possibility of functional recovery by neuronal replacement more and more practical (Yuan and Zhang 2024).

Creative approaches are being developed to improve the accuracy and safety of these regenerative techniques by using pre-patterned neuronal subtypes or biomimetic scaffolds (Giandomenico and Lancaster 2022).

The therapy of neurological illnesses is being changed by developments in circuit-specific interventions, neuromodulation, immune-targeted medications, and stem cell therapies. The combination of regenerative medicine with sophisticated neural circuit mapping technologies offers hopeful prospects for the restoration of brain function in formerly unmanageable circumstances. Realizing the full promise of these drugs will, therefore, depend on ongoing translational research, ethical issues, and rigorous clinical testing.

## Conclusion

Modern developments in neural circuit mapping tools including optogenetics, chemogenetics, and advanced neuromodulation techniques including transcranial magnetic stimulation (TMS) have changed our ability to precisely map, control, and alter separate brain circuits. By providing strong routes for the creation of circuit-specific therapy procedures, these tools have greatly improved our understanding of the fundamental ideas governing brain function and dysfunction.

While chemogenetics offers long-lasting, non-invasive modulation, optogenetics allows precise temporal control of neuronal populations, hence allowing scientists to examine the activities of particular neural circuits in health and illness. While new adaptive, closed-loop systems provide better therapeutic accuracy, the clinical use of neuromodulation technologies—including TMS and deep brain stimulation—is transforming the therapy of mental and neurological disorders.

Sophisticated drug delivery systems and stem cell therapy inclusion improve these developments. Stem cells not only have the ability to repair damaged neurons but also increase neuroplasticity and support neurological recovery. Innovative drug delivery systems including nanoparticle-mediated and focused ultrasound-guided ones provide exact control of brain circuits, hence improving treatment effectiveness and lowering systemic side effects.

Highly tailored therapies in neurology and psychiatry are being built on the combination of circuit mapping technologies, regenerative medicine, exact neuromodulation, and sophisticated drug delivery. These developments indicate a change toward drugs that aim the particular network dysfunctions driving brain diseases, thereby possibly boosting recovery outcomes and raising patients’ quality of life. Continuous innovation—including the integration of artificial intelligence, human brain organoid models, and wireless neuromodulation devices—is set to improve existing approaches and hasten the development of precision neuromedicine.

## Data Availability

No datasets were generated or analysed during the current study.
